# Comparison and correlation of in vitro and in vivo approaches for determining *Pseudomonas aeruginosa* bacteriophages activity

**DOI:** 10.1186/s12866-026-05289-w

**Published:** 2026-06-13

**Authors:** Pedro Henrique Takata, Giovana Nicolete Pereira, Jhonatan Macedo Ribeiro, Laura Pierobão, Gabriel Henrique Maximino Santos, Bruna Carolina Gonçalves, Pedro Ademário Lima e Silva, Mariana Marques Bertozzi, Rafael Reis de Rezende, Giarlã Cunha da Silva, Ruither Arthur Loch Gomes, Waldiceu A. Verri, Poliane Alfenas-Zerbini, Renata Katsuko Takayama Kobayashi, Gerson Nakazato

**Affiliations:** 1 Laboratory of Basic and Applied Bacteriology, Department of Microbiology, State University of Londrina, Londrina, Paraná Brazil; 2 Laboratory of Pain, Inflammation, Neuropathy and Cancer, Department of Immunology, Parasitology and General Pathology, Department of Pathological Sciences, State University of Londrina, Londrina, Paraná Brazil; 3 Institute of Biotechnology Applied to Agriculture (BIOAGRO), Department of Microbiology, Federal University of Viçosa, Viçosa, Minas Gerais Brazil

**Keywords:** Multidrug-resistant bacteria, Host range, Phage therapy, *Galleria mellonella*, Mice model, Peritonitis, Correlation coefficient

## Abstract

The rise of infections caused by multidrug-resistant bacteria has driven the search for alternative therapies, with *Pseudomonas aeruginosa* representing a major target. Among these alternatives, the use of bacteriophages (phage therapy) employs these viruses that specifically infect bacteria to fight infections. Although phage efficacy has been demonstrated both in vitro and in vivo, few studies have directly correlated these two dimensions. This study aimed to evaluate, compare, and correlate in vitro and in vivo phage activity. For this, three strictly lytic bacteriophages (ph9027, ph1461, and ph3678) were isolated and tested against 15 clinical isolates and three reference strains of *P. aeruginosa*. Their in vitro activity was assessed through Spot-Test, Efficiency of Plating, Local Virulence, and Virulence Index assays, followed by in vivo assays in *Galleria mellonella* larvae and Balb/C mice in a peritonitis model with bacteremia induction. Virulence Index and animal treatments were performed using a phage cocktail (1:1:1 ratio, 1.0 × 10⁹ PFU/mL). In vitro assays revealed varying phage activity, with the Virulence Index showing the strongest correlation with in vivo results in *G. mellonella* (rs = 0.7648). Larval treatment achieved up to 100% survival, significantly different (*p* < 0.05) from bacteria-only controls. In mice, phage therapy reduced bacterial loads by up to 3 logs in blood, spleen, peritoneal lavage, liver, and lung (*p* < 0.05) in comparison with control groups. Therefore, the present study, in addition to reiterating the safety and therapeutic efficacy of phage therapy in animal models, presents evidence that Virulence Index techniques, associated with the survival results of *G. mellonella* larvae, can be excellent tools for directing preclinical assays due to the strong statistical correlation observed between these data and those obtained in mouse experiments.

**Supplementary Information:**

The online version contains supplementary material available at 10.1186/s12866-026-05289-w.

## Introduction

First described by Frederick Twort in 1915 [1, 2] and used by Felix d’Herelle to treat children with bacterial dysentery in 1919 [3, 4] bacteriophages (phages) are viruses capable of infecting only bacteria, causing no effects on human cells [4]. However, despite the usage of phages against bacterial infections being older than antibiotic therapy, some questions like the discovery of penicillin itself [2, 5], contributed to the eclipse, except in the USSR, of bacteriophage usage. Nowadays, with the emergence of multidrug-resistant (MDR) bacteria, emphasized by organizations of global importance such as the U.S. Centers for Disease Control and Prevention (CDC) and the World Health Organization (WHO) [6, 7], culminated in an increase in research on new antimicrobials as therapeutic alternatives. Among them, phage therapy stands out, increasingly gaining ground worldwide as an antimicrobial therapy. In this sense, due to its high clinical relevance in the described global scenario and its established history of successful bacteriophage applications as a therapeutic mechanism, the opportunistic pathogen *Pseudomonas aeruginosa*—a Gram-negative bacillus belonging to the concerning ESKAPEE group (acronym for *Enterococcus faecium*,* Staphylococcus aureus*,* Klebsiella pneumoniae*,* Acinetobacter baumannii*,* Pseudomonas aeruginosa*,* Enterobacter sp.*, and *Escherichia coli*) and frequently associated with infections in cystic fibrosis patients—was selected as the target bacterium for this study [8, 11]. This bacterium possesses extensive machinery for developing resistance to conventional antimicrobials, further reinforcing the need to seek therapeutic alternatives [12].

Nevertheless, although the efficacy of bacteriophages as therapy has already been validated even in clinical scenarios and applications [13, 15], an important question remains within the bacteriophage scientific community: how is it possible to establish a concrete link between in vitro results and in vivo results? This issue is raised because there are numerous in vitro techniques that are supposedly capable of detecting the therapeutic activity of bacteriophages [16, 19]. However, such results generated from studies employing these techniques have, for the most part, not sought to extrapolate in vitro* findings* to the in vivo context up to that point, creating a disconnect between these environments. Thus, the present work sought to delve into this nebulous question that still permeates phage therapy, aiming to clarify the best directions and reliable parameters to establish this bridge between in vitro and in vivo in the development of phage therapies. For this purpose, we applied in vitro (Spot Test, EOP, Local Virulence and Virulence Index) and in vivo (*Galleria mellonella* and mouse survival assay) methods and analyzed the correlation between the results to determine if the in vitro methods can reasonably predict in vivo efficacy.

## Methods

### Bacterial isolates and bacteriophages

A total of 15 *Pseudomonas aeruginosa* isolates and three reference strains were used in this study. Standard strain ATCC 9027 and 27,853 was purchased from the American Type Culture Collection (ATCC, Gaithersburg, MD, United States) whereas the clinical isolates were provided from University Hospital of Londrina bacterial bank. The number and isolation source of bacteria used in this study was determined by the availability of these bacteria provided by the University Hospital of Londrina during the period of the study. More information about these isolates were provided in Supplementary Materials. Bacteriophages were isolated and purified from liquid and solid samples from commercial poultry farms, slaughterhouses, and hospital sewage from University Hospital of Londrina (Paraná state, Brazil), as described previously [20, 21]. Briefly, liquid samples were initially centrifuged at 5000 × g for 5 min, and the supernatant was collected in 15 mL conical tubes. To this supernatant, 5% (v/v) chloroform was added and mixed by inversion for 5 min. Subsequently, another centrifugation was performed under the same conditions as before, and the supernatant was collected again and used for bacteriophage propagation. For solid samples, 5 g of each sample was added to 10 mL of SM buffer in 15 mL conical tubes and mixed by inversion for 10 min. These tubes were centrifuged at 5000 × g for 5 min, and the supernatant was collected and processed in the same manner as the liquid samples. Subsequently, in 50 mL conical tubes, 10 mL of the supernatant was mixed with 2x concentrated LB broth supplemented with 20 mM CaCl₂, and 100 µL of each bacterial sample in exponential growth phase was added to this mixture. The tubes were then incubated for 20 h at 37 °C with shaking at 120 rpm. After this period, 5% (v/v) chloroform was added to the tubes, mixed by inversion for 5 min, and the tubes were centrifuged at 5000 × g for 5 min. The supernatant was collected and stored at 4 °C for subsequent tests.

Preliminarily, phage activity detection was performed as described previously with the Streak-Spot Test [20, 21] by spotting 5 µL from each lysate onto streaks of the bacteria in Luria-Bertani (LB) agar plates supplemented with 10 mM CaCl_2_. Then, the presence of bacteriophages was confirmed by the observation of an inhibition of bacterial growth in the spotted zone. The bacteria against which the phage showed full and clear inhibition were arbitrarily defined as the phage’s bacterial propagation host.

### Bacteriophage host range determination assays

Bacteriophages host range were determined by Spot-Test (ST), Efficiency of Platting (EOP), Local Virulence (v_1_) and Virulence Index (V_P_). Spot-Test was performed as described previously [17, 22]. For the results, the formation of inhibitions zones was classified from “0” (no inhibition zone) to “+++” (clear inhibition zone with no growth of bacterial colonies). All the isolates were tested, and their results were plotted using GraphPad Prism v. 9.1.1. in heatmap graphics. Efficiency of Platting was performed as the same for titration of bacteriophages [17]. Relative Efficiency of Plating was calculated by the division of the average PFU on the tested bacteria sample by the average PFU on the host bacteria (theoretically, the bacterium with the maximum plaque counts). The EOP value for the phage-bacteria combination was classified as “High” for ratios ≥ 0.5, “Medium” for ratios ≥ 0.1 and < 0.5, “Low” for values > 0.001 and < 0.1, and “Inefficient” for ratios ≤ 0.001 [21, 22] and were plotted using GraphPad Prism v. 9.1.1. in heatmap graphics. Local Virulence and Virulence Index were performed as described previously [21, 23, 24]. Local virulence and Virulence index score were classified as “High” for values > 0.5, “Medium” for ratios ≥ 0.2 and ≤ 0.5, “Low” for values ≥ 0.001 and < 0.2, and “Inefficient” for ratios < 0.001 and were plotted using GraphPad Prism v. 9.1.1 in heatmap graphics.

### *Galleria mellonella* phage therapy assay

*Galleria mellonella* phage therapy assays were performed as described previously [10, 25, 26]. Only cream-colored 6th instar larvae, weighing 200–250 mg and with no injury marks, were used. First, larvae were separated into negative control (PBS-only) (pH 7.4), bacteria positive control (bacteria-only), phage cocktail positive control (phage cocktail-only), and treatment (cocktail-bacteria) groups of 10 larvae each one. At first, the bacteria positive control and treatment group were inoculated with 10 µL of overnight cultures of *P. aeruginosa* isolates (1.0 × 10^4^ CFU**/**mL), and the negative and phage cocktail positive control group were inoculated with 10 µL of PBS. After 2 h, treatment and phage cocktail positive control group were inoculated with 10 µL of bacteriophage cocktail formulation (titer varying from MOI 10^4^ to 10^5^), and the negative and bacteria positive control group were inoculated with 10 µL of PBS. Finally, all larval groups were placed into Petri dishes and incubated at 37 °C during 48 h, and were recorded as dead when they did not move in response to touch and in presence of full body melanization. All larvae inoculations were performed using a Hamilton syringe into the last left proleg.

### Mouse phage therapy assay

Mouse survival tests were performed using a peritonitis model for up to 7 days with female Balb/C mice, approximately 6–8 weeks old, as previously described [27, 29], with adaptations, utilizing *Pseudomonas paraeruginosa* strain ATCC 9027 (Pa9027) and Pa49626, a clinical *P. aeruginosa* isolate. All mice used in this study were provided by the Central Animal Facility of the Biological Sciences Center at the State University of Londrina (UEL), upon presentation of the approval protocol number. 075/2024, granted by the Ethics Committee on the Use of Animals of the State University of Londrina (CEUA-UEL).

Initially, a dose–response test was conducted to obtain LD_50_. The animals were divided into four groups and inoculated with different concentrations of bacteria. The animals were euthanized and evaluated at the following time points: 24 h, 3 days, 5 days, and 7 days (*n* = 5 per time point). The groups received bacterial inoculum of 9.0 × 10⁸, 6.0 × 10⁸, and 3.0 × 10⁸ CFU/mL, along with a PBS (pH 7.4) vehicle control group (*n* = 20 per group). At each time point, bacterial kinetics in the animals were assessed by collecting and plating samples on nutrient agar from blood (via cardiac puncture) and spleen. The final volume of all inoculations was 100 µL. Animals exhibiting advanced morbidity were euthanized before the scheduled endpoint. After defining LD_50_, the mice were subjected to a new assay involving a double-blinded bacteriophage treatment to evaluate the efficacy of phages in combating Pa9027 and Pa49626 infection. For this purpose, for every bacterium, the animals were divided into three new experimental groups: (1) negative control group (phage only), (2) positive control group with the LD_50_ of the bacterium, (3) treatment group with phages in animals infected with the LD_50_ (*n* = 20 per group). In this experiment, no time points for biological sample collection were established (samples were collected only on the final day of analysis, which was determined based on the dose–response test). Phage treatments were administered at a concentration of 1.0 × 10⁹ PFU/mL, delivered via intraperitoneal injection in a final volume of 100 µL, 2 h after bacterial inoculation and every 12 h following the initial treatment until euthanasia for biological sample collection (peritoneal lavage, spleen, lungs, blood, and liver). The tissues were used for the quantification of bacteria and bacteriophages, as well as for measuring Nitro-Blue Tetrazolium (NBT), N-acetyl-β-glucosaminidase (NAG), and myeloperoxidase (MPO). Prior to any procedure, the animals were anesthetized via intraperitoneal administration of xylazine hydrochloride (10 mg/kg). All euthanasia procedures were performed by administering an overdose of 5% isoflurane in O₂ via inhalation.

### Bacteriophage genomic DNA extraction

Phage DNA was extracted from 1 mL of purified virus stock using the phenol-chloroform method as described previously [21, 30]. Phage suspensions was first treated with DNase I (1 µg/mL) and RNase (10 mg/mL) (Promega) for 1.5 h at 37 °C, and then the nuclease was inactivated with 50 µL of EDTA. After this, 3 µL of proteinase K (20 mg/mL) and 50 µL of sodium dodecyl sulfate (SDS) (10%) were added, and the mixture was incubated at 65 °C for 1 h. Then, 1 mL of phenol was added and centrifuged for 5 min at 8,000 × g. Therefore, 1 volume of phenol-chloroform (1:1) and subsequently 1 volume of chloroform were separately added to the aqueous phase and centrifuged for 5 min at 8,000 × g. Then, 2 volumes of absolute ethanol were added, incubated at -20 °C for 30 min, and centrifuged for 20 min at 21,000 × g. Finally, the pellet was washed twice with 70% ethanol (5 min at 8,000 x g), air-dried briefly, and reprecipitated in deionized water.

### Bacteriophage genome sequencing and bioinformatics analysis

Phage sequencing was performed using Illumina MiSeq. Quality of sequenced reads was verified using FastQC (v. 0.11.9), and trimming was performed using Trimmomatic (v. 0.39) [31]. SPAdes (v. 4.0.0) [32] was used for genome assembly and CheckV (v. 1.0.3) [33] was used for checking a single viral contig and the completeness of the assembled viral genome. For multiple phage genome alignment, DigAling (v. 2.0) [34] was used, and for assessing similarity between phages, VIRIDIC (v. 1.1) [35] and VIPTree (v. 4.0) [36] were used. For genome annotation, Prokka (v. 1.14.6) [37] and Pharokka (v. 1.3.2) were used [38]. PhaBOX2 (v. 2.1.13) was used for phage lifestyle prediction [39].

### Statistical analysis

*G. mellonella* larvae and mice survival were plotted into Kaplan-Meier curves, and log-rank (Mantel-Cox) statistical test was performed to verify significant (*p* < 0.05) survival differences between the groups. Bacterial, bacteriophage, and NAG, NBT and MPO levels in the different groups are presented as mean ± SD, and statistical significance was assessed using one-way ANOVA or unpaired t-test (*p* < 0.05).

## Results

### Bacteriophage isolation, genomic analysis, and host range determination

**Fig. 1:**
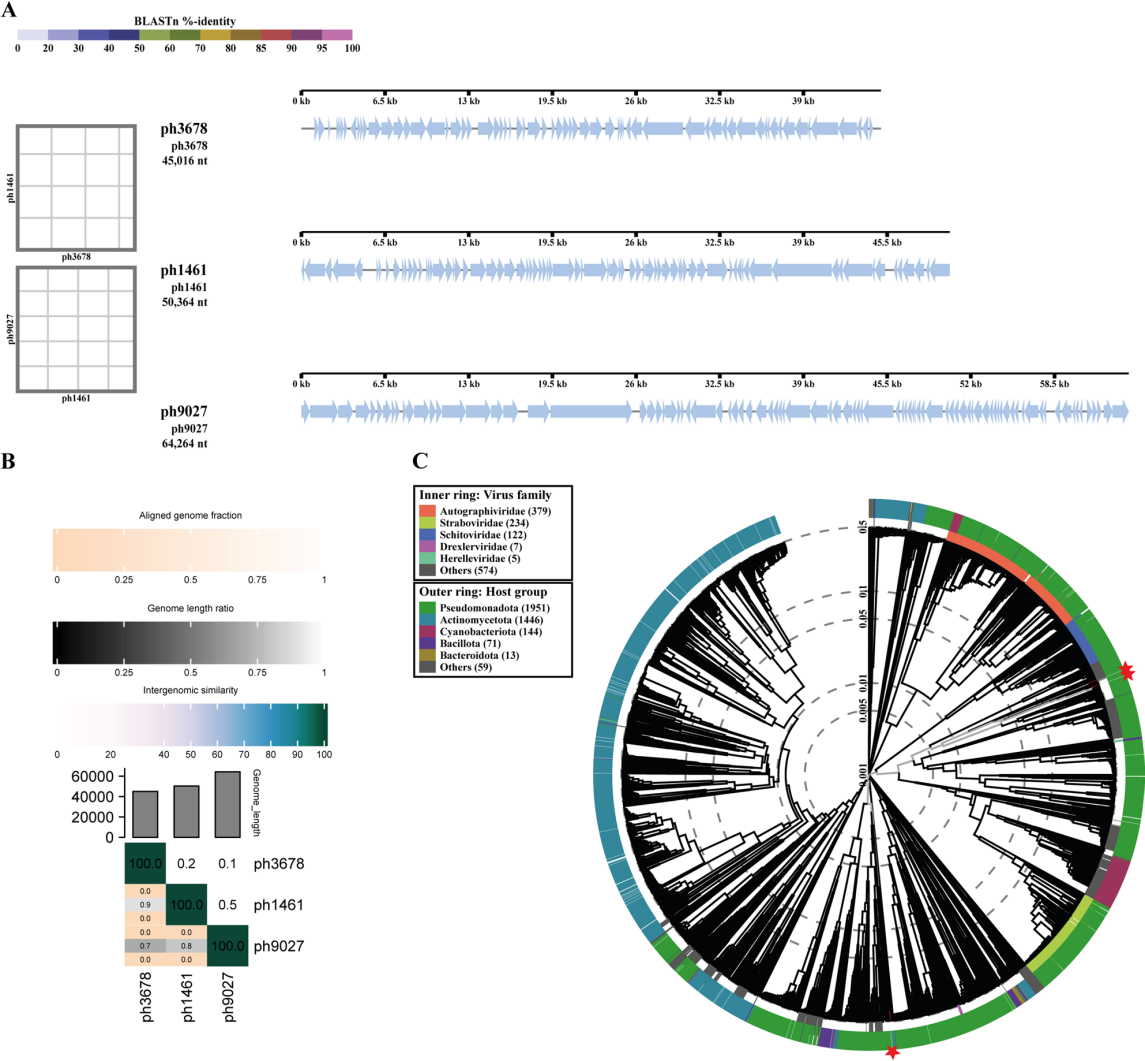
Genome analysis of phages ph3678, ph1461, and ph9027. **A** Multiple genome alignment of the three genome phages using DiGAlign (v. 2.0). The individual phage annotation and description are shown in Supplementary Materials. **B** Heatmap of intergenomic similarities and alignment indicators for the three phages constructed using VIRIDIC (v. 1.1). In the right half, color intensity is based on the intergenomic similarity of phage genomes, and the numbers represent the similarity values for each pair. In the left half, three indicator values are represented for each genome pair, in the order from top to bottom: aligned fraction genome 1 (for the genome found in the row), genome length ratio (for the genome pair), and aligned fraction genome 2 (for the genome found in the column). **C** Proteomic tree of viral genome sequences based on genome-wide sequence similarities computed by tBLASTx constructed using VIPTree (v. 4.0). Red stars indicate, in a clockwise direction, phages ph1461, ph3678, and ph9027

Three bacteriophages were successfully isolated from hospital sewage (Fig. 1), with no genes related to lysogeny, resistance, or virulence detected in their genomes. Thus, all bacteriophages were classified as strictly lytic. According to our analyses, phage ph9027 has a 45,016 bp genome encoding 98 CDS and is classified within the genus *Kochitakasuvirus*. Phage ph1461 has a 50,364 bp genome with 92 CDS and belongs to the genus *Paundecimvirus*, whereas phage ph3678 possesses a 45,016 bp genome encoding 92 CDS and is classified within the genus *Bruynoghevirus*. Their names were arbitrarily designated according to their respective propagation hosts Pa9027, Pa1461, and Pa3678). The functional genomes image of these three phages and gene annotation were provided in the Supplementary Materials.

**Fig. 2:**
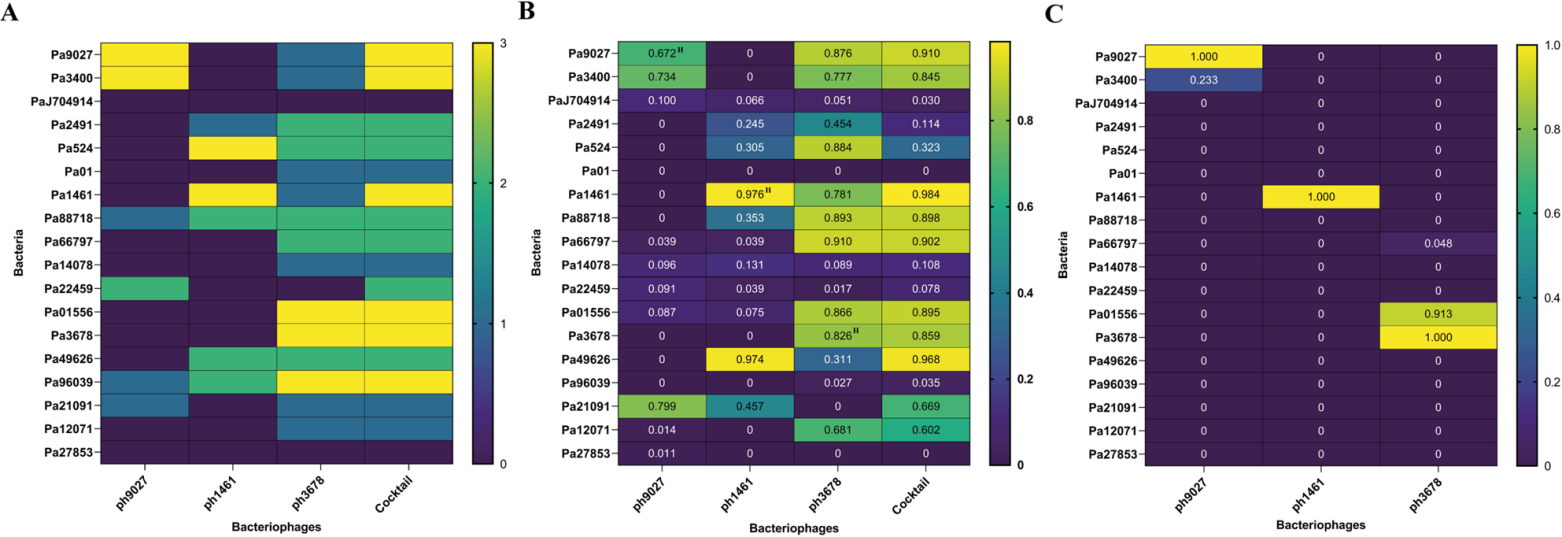
Characterization of the bacteriophage host range according to (**A**) Spot Test, (**B**) Local Virulence, and (**C**) Efficiency of Plating. Colors ranging from dark blue to light yellow indicate the degree of phage activity in the respective tests, with bluer shades representing poorer activity and yellower shades indicating better activity. As it is a discrete variable, the activity level in the ST assay is represented by 4 fixed colors (values assigned from 0 to 3), while the values from the other tests, being continuous variables, are presented as a color spectrum. The value obtained for the test against the host bacterium of each phage is superscripted with the letter “H”

These phages were combined in equal proportions (1:1:1), yielding a final concentration of 1.0 × 10⁹ PFU/mL to formulate the phage cocktail and demonstrate activity against up to 89% (16/18) of the tested bacteria. Overall, ph3678 exhibited the broadest host range among the others, characterizing it as a generalist phage. It also showed high performance across all assays designed to assess this parameter, whereas the other two phages displayed a more restricted host range, indicating a specialist profile [18] (Fig. 2) (Table 1). Such conclusions were drawn based on the bacterial panel available for this study, which, however, could change if a different panel were used.

**Table 1:** Percentage of bacterial isolates sensitive to bacteriophages across different host range techniques

Technique	Bacteriophage			
	ph9027	ph1461	ph3678	Cocktail
Spot Test	33% (6/18)	33% (6/18)	83% (15/18)	89% (16/18)
Efficiency of Platting	11% (2/18)	6% (1/18)	17% (3/18)	-
Local Virulence	56% (10/18)	61% (11/18)	83% (15/18)	89% (16/18)
Virulence Index	-	-	-	61% (11/18)

### *Galleria mellonella* phage therapy survival assay

**Fig. 3:**
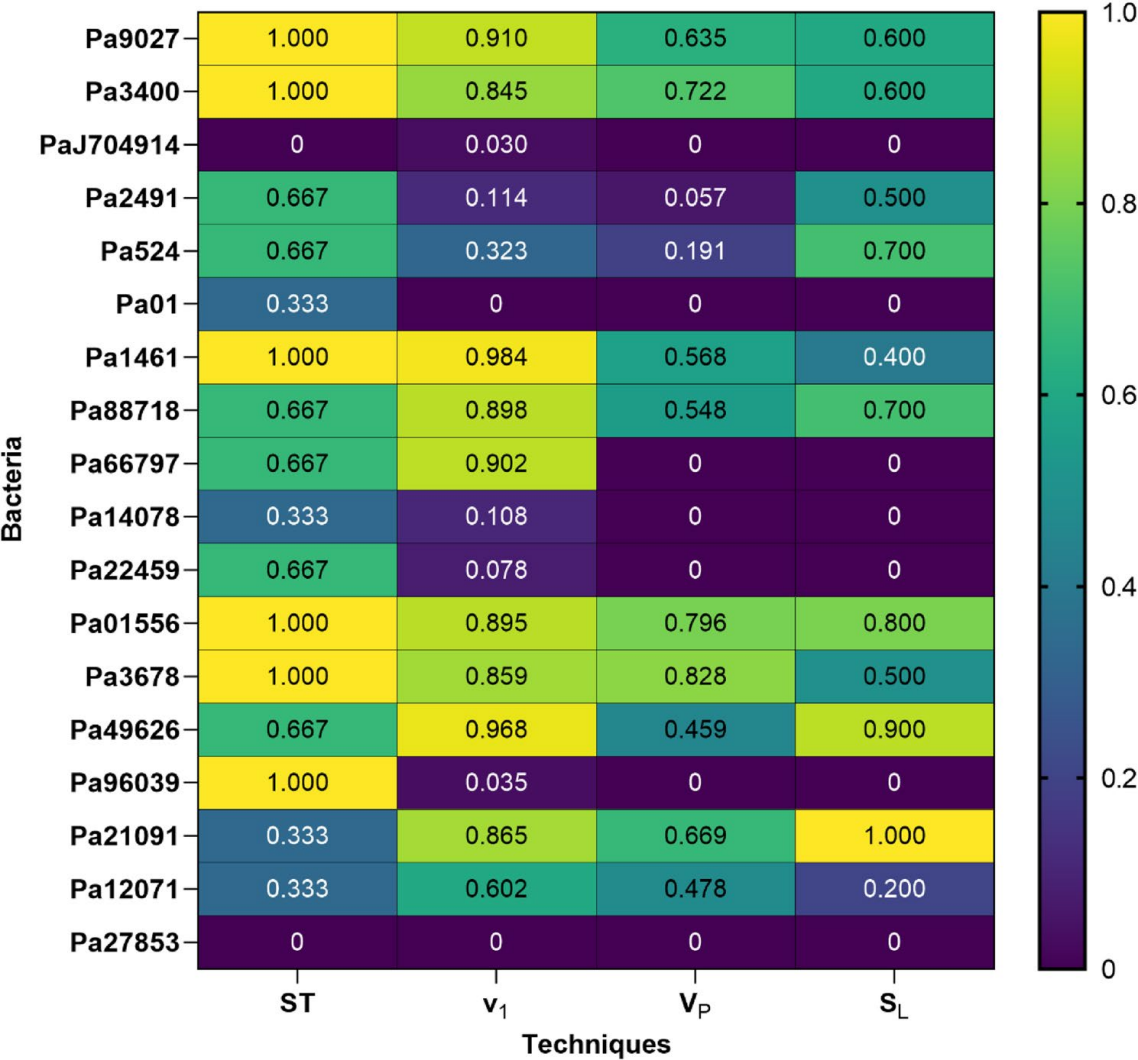
Characterization of the host range of the bacteriophage cocktail (1:1:1 mixture of individual phages) using different in vitro techniques (ST, v_1_, and V_P_), along with the survival rate of *G. mellonella* larvae treated with the cocktail (S_L_). Colors ranging from dark blue to light yellow indicate the degree of phage activity in the respective tests, with bluer shades representing poorer activity and yellower shades indicating better activity. As it is a discrete variable, the activity level in the ST assay is represented by 4 fixed colors (values assigned from 0 to 3), while the values from the other tests, being continuous variables, are presented as a color spectrum

The bacteriophage cocktail was able to provide no toxicity and up to 100% larval survival in the treatment group, showing a statistically significant difference compared to the infection control group (bacteria only) in all cases where survival exceeded 0% (*p* < 0.05). All the results of the in vitro assays with the phage cocktail, as well as the survival rates of *Galleria mellonella* larvae (S_L_), are presented in the heatmap (Fig. 3) and observed levels across the bacterial strains tested, was categorized as inefficient (0 < S_L_ ≤ 0.1), low (0.1 < S_L_ ≤ 0.3), moderate (0.3 < S_L_ < 0.6), or high (S_L_ ≥ 0.6). These classifications are summarized in Table 2. Based on the comparison of the ST, v_1_, and V_P_ values with S_L_, we hypothesize that there may be a strong correlation between the results obtained from V_P_ and S_L_, as both parameters appear to follow a similar pattern—that is, when one value is low, the other also tends to be low, and vice versa—even though their numerical results are not exactly identical.

**Table 2:** Comparison of cocktail activity levels across different host range (HR) techniques

Technique	Percentage of activity			
	Inefficient	Low	Moderate	High
Spot Test	11% (2/18)	22% (4/18)	33% (6/18)	33% (6/18)
Local Virulence	11% (2/18)	28% (5/18)	6% (1/18)	56% (10/18)
Virulence Index	39% (7/18)	11% (2/18)	11% (2/18)	39% (7/18)
Larvae Survival	39% (7/18)	6% (1/18)	17% (3/18)	39% (7/18)

**Fig. 4:**
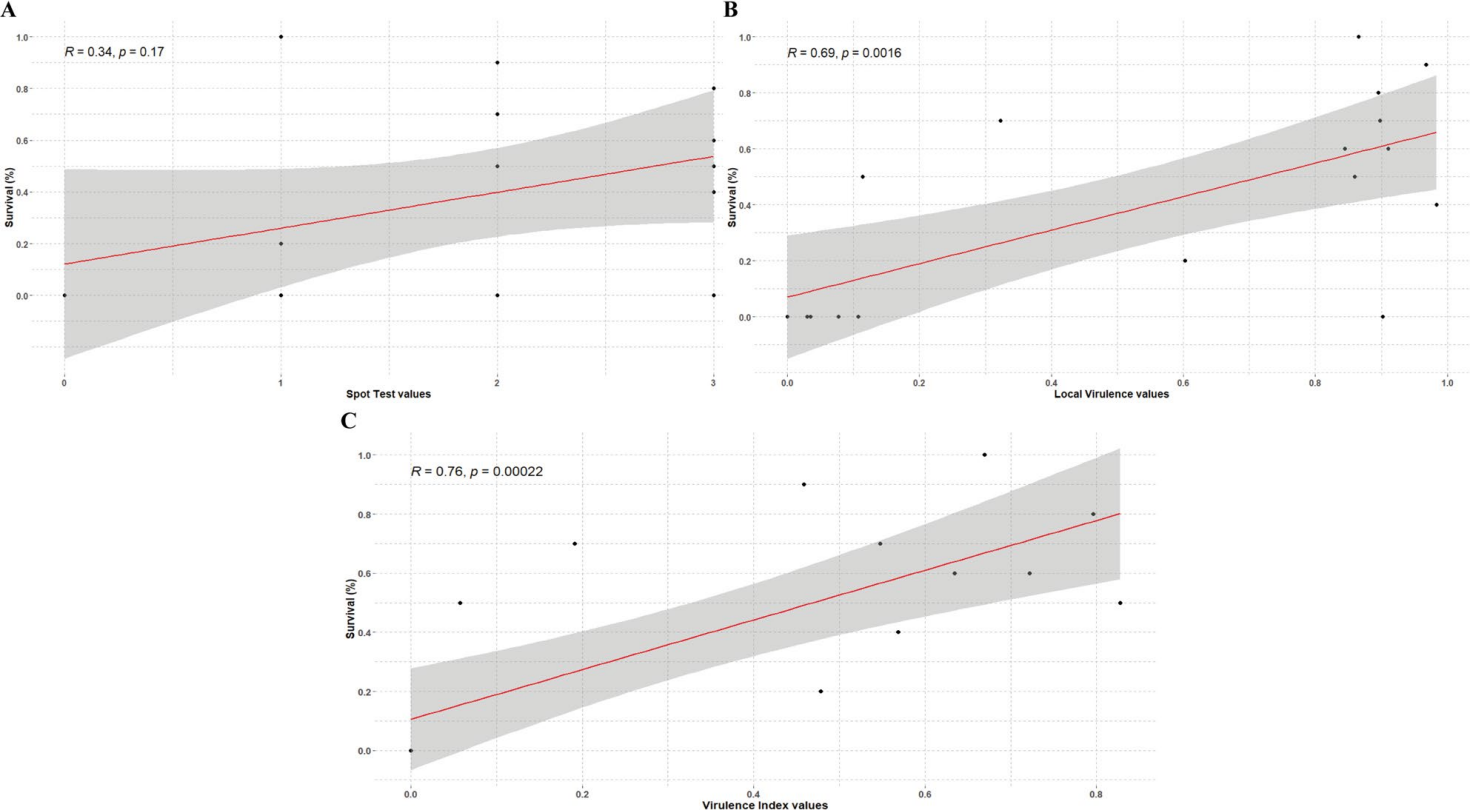
Results and values from the (**A**) Local Virulence, (**B**) Spot Test, and (**C**) Virulence Index assays compared with the survival rate of G. mellonella larvae treated with the cocktail. Correlation coefficients were calculated using Spearman’s test for the comparison between ST and survival (*p* = 0.1669, *r* = 0.3404), and Pearson’s test for the comparisons between v1 and survival (*p* = 0.0016, *r* = 0.6892) and between VP and survival (*p* = 0.0002, *r *= 0.7648)

To assess the correlation between all in vitro assays (ST, v_1_, and V_P_) and the in vivo survival rates of *G. mellonella* larvae (S_L_), statistical correlation analyses were performed. Spearman’s test was applied to evaluate the association between ST and S_L_, given the ordinal nature of the ST response variable (Fig. 4B). In contrast, Pearson’s test was employed to analyze the correlations between v_1_ and S_L_, as well as V_P_ and S_L_, considering the continuous nature of the response variables in the v_1_ and V_P_ assays (Fig. 4A and C). The correlation analyses revealed a coefficient of 0.3040 (p = 0.1669) for ST and S_L_, indicating a weak correlation. For v_1_ and S_L_, a coefficient of 0.6892 (p = 0.0016) was observed, consistent.

with a moderate correlation. Finally, V_P_ and S_L_ showed a coefficient of 0.7648 (p = 0.0002), indicative of a strong correlation, as expected. [40].

### Mice phage therapy survival assay

**Fig. 5:**
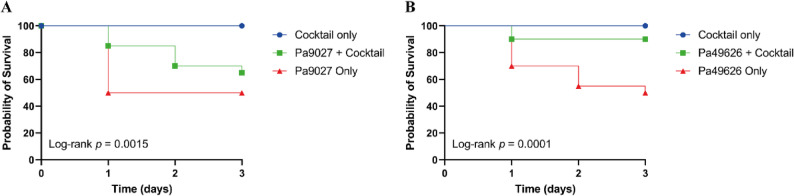
Survival curves of mice infected with 3.0 × 10^8^ CFU/mL of (**A**) Pa9027 and (**B**) Pa49626 and treated with 1.0 × 10^9^ PFU/mL

According to the dose–response assay, the lethal dose 50 (LD₅₀) determined for the tested bacterium was 3.0 × 10⁸ CFU/mL, which was subsequently used as the inoculum in the bacteriophage treatment assay. In the experiment conducted with Pa9027, the mortality rate out of 10 mice per group was 10 mice in the positive control group (bacteria plus vehicle), 7 mice in the treatment group, and none of the animals from the negative control group (phage only) (Fig. 5A). To better understand the mechanisms of phage cocktail action, there are some important parameters. CFU recovery accounts to understand if increased survival rate might be related to improved bacterial clearance. As the primary bacterial infection foci was the peritoneal cavity, peritonitis evolves to sepsis when bacteria reach the blood stream and disseminates to other tissues.

**Fig. 6:**
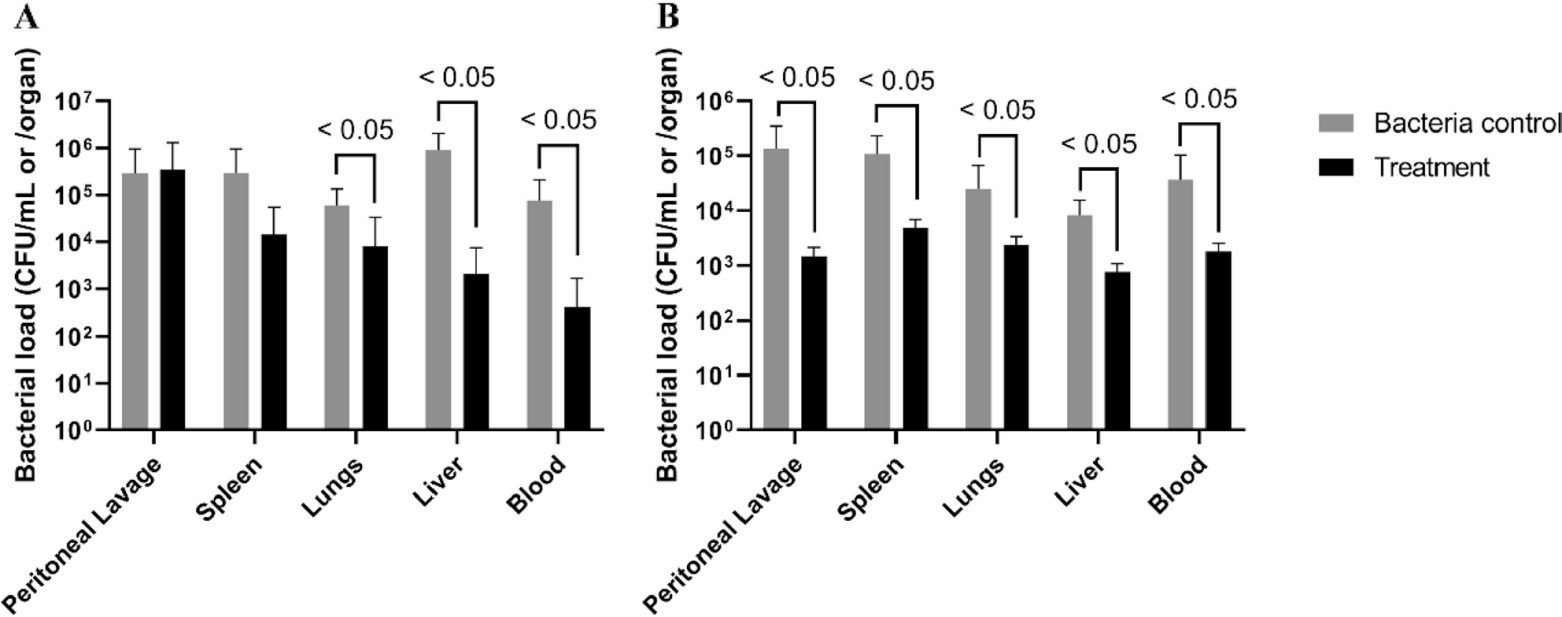
Bacterial quantification in the infection treatment model induced by (**A**) Pa9027 and by (**B**) Pa49616 in peritoneal lavage, spleen, lung, blood, and liver. The infection dose for both bacterial strains was 3.0 × 10⁸ CFU/mL, and the treatment dose was 1.0 × 10⁹ PFU/mL of the phage cocktail

**Fig. 7:**
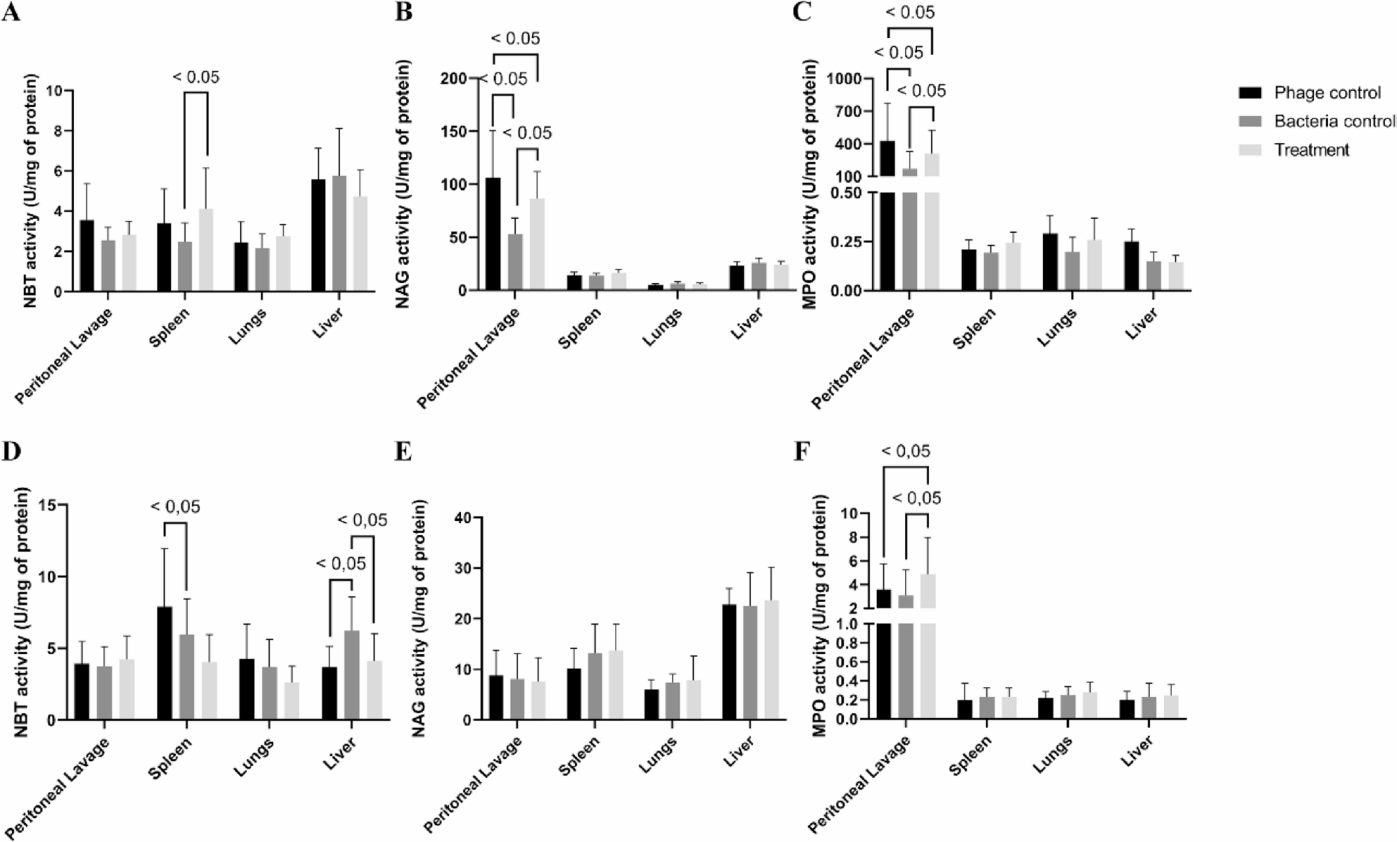
Levels of (**A**) NBT (**B**) NAG and (**C**) MPO in the infection treatment model induced by Pa9027, and (**D**) NBT (**E**) NAG and (**F**) MPO in the infection treatment model induced by Pa49626. Peritoneal lavage, spleen, lungs and liver were used for these titrations

Therefore, reduction of CFU in the blood and organs indicates that survival rates will increase. Leukocyte recruitment is essential to control bacterial infection. Macrophages (NAG assay) and macrophages/ neutrophils (MPO assay) are important to be present in the peritoneal cavity to produce reactive oxygen species such as superoxide anion (NBT assay) to kill bacteria [16, 41]. Regarding bacterial loads in the collected tissues, Pa9027 induced a significant reduction in all organs analyzed, except for the peritoneal lavage (Figs. 6A). In contrast, in the experiment performed with Pa49626, the mortality rate out of 10 mice was 10 mice in the positive control group (bacteria only), 2 mice in the treatment group, and none of the animals in the negative control group (phage only) (Fig. 5B). Regarding bacterial loads in the collected tissues, Pa49626 treatment induced a significant reduction in all organs analyzed (Figs. 6B). control (bacteria only), with statistically significant differences (*p* < 0.05) for infections induced with both bacterial strains. Phages were detected in all tissues in phage control group and only in spleen in treatment group. In addition to these data, the levels of NBT, NAG, and MPO found in the mouse tissues demonstrate important biological findings, enhancing the description of the course of both assays with the different bacteria (Fig. 7). The NBT assay quantitates superoxide anion production, NAG assesses indirectly the macrophage counts and MPO the neutrophil/ macrophage counts [42]. The results of these markers together with the microbiological data allow for some consideration. In both infection cases (Pa9027 and Pa49626), there was a higher neutrophil activation rate at the primary infection site, the peritoneum, compared to the other analyzed tissues, as indicated by the exacerbated detection of MPO in that region (Fig. 7C and F). A relatively higher macrophage activation rate was also observed in the peritoneum, indicated by the exacerbated detection of NAG in that region, but again in greater proportions in the infection caused by Pa9027 (Fig. 7B and E). Finally, a relatively similar amount of oxidative metabolism products was produced in both scenarios, as indicated by the NBT levels (Fig. 7A and D).

The bacteriophage cocktail was effective in reducing both mortality and bacterial burden without causing toxicity, achieving decreases of up to 3 logs in the treatment group compared with the positive control (bacteria only), with statistically significant differences (*p* < 0.05) for infections induced with both bacterial strains. Phages were detected in all tissues in phage control group and only in spleen in treatment group. In addition to these data, the levels of NBT, NAG, and MPO found in the mouse tissues demonstrate important biological findings, enhancing the description of the course of both assays with the different bacteria (Fig. 7). The NBT assay quantitates superoxide anion production, NAG assesses indirectly the macrophage counts and MPO the neutrophil/ macrophage counts [42]. The results of these markers together with the microbiological data allow for some consideration. In both infection cases (Pa9027 and Pa49626), there was a higher neutrophil activation rate at the primary infection site, the peritoneum, compared to the other analyzed tissues, as indicated by the exacerbated detection of MPO in that region (Fig. 7C and F). A relatively higher macrophage activation rate was also observed in the peritoneum, indicated by the exacerbated detection of NAG in that region, but again in greater proportions in the infection caused by Pa9027 (Fig. 7B and E). Finally, a relatively similar amount of oxidative metabolism products was produced in both scenarios, as indicated by the NBT levels (Fig. 7A and D).

## Discussions

The determination of bacteriophage host range (HR) remains a central debate in phage research [16, 19]. This parameter is critical for phage therapy, as demonstrating phage activity by at least one method is an essential requirement for further viral characterization and therapeutic use. Although phages are often described as highly specific entities, they may display broad spectra of activity, either within a bacterial species or across species in the case of polyvalent phages [43]. Such breadth of host recognition—driven by the ability to bind multiple receptors—has been discussed as an adaptive evolutionary strategy in diverse bacterial communities [43, 45]. However, different methodological approaches can yield conflicting HR results. The most frequent discrepancies occur between Spot-Test (ST) and Efficiency of Platting (EOP) [22, 46, 48]. In our study, HR values obtained by ST were considerably higher than those by EOP (e.g., phage ph3678: 89% vs. 17%). This divergence stems from the parameters each assay detects: ST identifies any growth inhibition zone, while EOP only confirms productive infections through plaque formation. Consequently, ST often overestimates activity, as inhibition zones may result not only from productive infections but also from abortive infections [49], lysis from without [50], or bacteriocin residues [22, 48]. Conversely, EOP may underestimate activity, as plaque formation depends on multiple factors beyond replication capacity [21, 51]. Thus, ST may generate false positives, while EOP risks false negatives. Importantly, even non-productive mechanisms (abortive infections, lysis from without, bacteriocins) may still provide antibacterial effects relevant to phage therapy [52].

Other assays add further complexity. Local Virulence (v_1_), a broth-based method, often yields activity profiles closer to ST than to EOP but can be influenced by medium-dependent phage–bacteria interactions and the emergence of resistant mutants [20] and evaluates only one MOI. Similarly, the Virulence Index (V_P_) approach tends to give intermediate results, lower than ST/v_1_ but higher than EOP. This is partly because V_P_ integrates outcomes across a broad range of multiplicities of infection (MOIs, 1–10⁻⁷), favoring phages that remain effective even at low concentrations. Henry et al., for example, when testing the host range of bacteriophage PhiKZ, observed a high efficiency of plating (1.2) relative to the PAK-lumi strain and good broth kinetics at a multiplicity of infection (MOI) of 0.001 (a parameter like what is referred to here as Local Virulence). However, when they proceeded to in vivo experiments in mice, even with an MOI of 0.1, the treatment was not effective, showing efficacy only at an MOI of 20. This result clearly demonstrates that the relative efficacy determined based on a single MOI, especially in broth assays, could be extremely risky for selecting therapeutic conditions for in vivo testing, which further supports the use of the Virulence Index parameter [53, 54].

By avoiding overestimation from high-MOI effects, V_P_ may represent a more stringent parameter for assessing true lytic capacity [55]. In summary, no single assay already described can fully capture HR: ST is sensitive but may overestimate, EOP is stringent but may underestimate, v_1_ is influenced by liquid culture dynamics, and V_P_ provides a more balanced estimate. Therefore, while ST is useful for preliminary screening, EOP remains inadequate as a sole criterion. V_P_, by minimizing false positives and negatives, emerges as a promising in vitro tool.

Such discussion creates a direct clash with a practice that has been gaining increasing traction in the bacteriophage community: the prediction of phage host range using machine learning. Although fantastic and often complex models of this nature have been developed, most of these systems are fed with binary information about bacteriophages (such as “lyses” or “does not lyse”) or with broth culture curves that consider only a single multiplicity of infection [56, 58]. Thus, given that researchers working in the field of phage therapy are always aiming at in vivo applications, using models that are fed with parameters that do not consider the full complexity of phage biology may create a therapeutic fallacy. That is, there may be a prediction at the *in silico–*in vitro level, but this bridge does not extend to the in vivo context, which is the destination that the vast majority of phage research groups aim to reach.

Moving forward to the in vivo assays in *G. mellonella* larvae, this study found highly positive results regarding the control of *P. aeruginosa* infection. This highly positive outcome is consistent with the extensive body of literature supporting the use of this alternative model to evaluate the efficacy of bacteriophage-based bacterial infection control [10, 25, 26, 59, 67]. However, although promising, the results obtained—depending on the in vivo parameter compared—may represent a confounding factor, as there is no unique convergence of these parameters with the observed survival rates (S_L_).

**Fig. 8:**
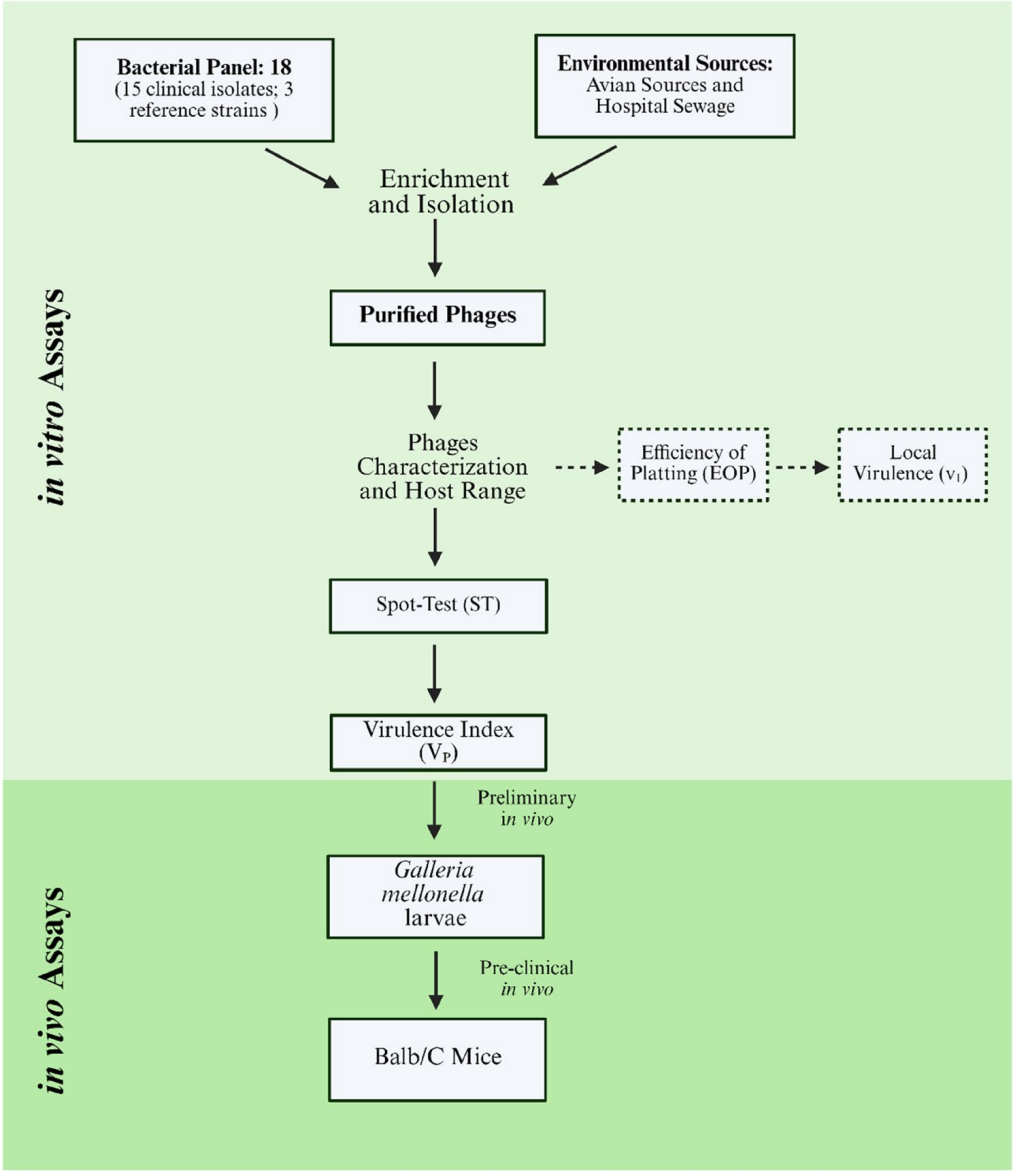
Proposed pipeline in this work for determining the therapeutic activity of bacteriophages, encompassing assays from in vitro techniques to preclinical in vivo studies

Such variability complicates therapeutic evaluation, as even basic validation may not reliably predict outcomes. Ideally, assays should minimize methodological bias, balance sensitivity and specificity, and better reflect in vivo performance. In this context, our data indicates that the Virulence Index (V_P_) assay correlated most closely with *G. mellonella* survival (S_L_). The correlation was both qualitative—all V_P_ inefficiencies aligned with poor S_L_ outcomes—and quantitative, as confirmed by Pearson’s correlation coefficient (r_s_). Thus, while still a preliminary in vivo approach for only possessing innate immunity composed of macrophage-like cells and other simple immune components, and lacking adaptive immunity [68, 70], the *G. mellonella* model, which is gaining attention in recent works [59, 62, 65, 71, 72], provides a practical bridge between in vitro assays and more complex in vivo systems such as murine models. Within this pipeline, the V_P_ assay stands out as the most reliable in vitro predictor to guide subsequent in vivo experimentation (Fig. 8).

The present work reaches its peak with the results of the mouse survival assay. Regarding treatment efficacy, phage cocktail induced survival rates reaching 30% (3 out of 10 animals at risk) for infections induced by the strain Pa9027 and 80% (8 out of 10 animals at risk) for those infections caused by the strain Pa49626. The detection of bacteriophages in all tissues of the cocktail control group confirms their ability to disseminate and accumulate in the peritoneal lavage, spleen, lungs, blood, and liver, as previously reported. Phages have also been described in the kidneys and heart in earlier studies [54, 73].

An important observation was the absence of detectable bacteriophages in most tissues of the treatment group (except the spleen), which is consistent with prior findings suggesting that the simultaneous presence of bacteria and phages in the host may accelerate immune clearance due to enhanced stimulation of the immune system [54, 74, 75, 76]. The persistence of phages in splenic tissue aligns with their known longer half-life in this organ compared with other tissues [10, 54, 73, 77]. The peritoneal cavity is the primary foci of infection in the present experimental condition. The phage cocktail presented a better effect against Pa49626 as we can observed in the survival rate, but also in the reduction of CFU in the peritoneal cavity. It is likely that the phage cocktail by reducing the infection in the primary infection foci reduced the bacterial infection lethality with a more prominent activity against Pa49626. In all other tissues (e.g. spleen, lung, blood and liver) the effect of the phage cocktail was similar against Pa9027 and Pa49626. Regarding the presence of macrophages and neutrophils in the tissues as well as superoxide anion production, there was no significant difference among groups in all tissues. This is likely because the activity of the phage cocktail is not dependent on interfering with the immune response, but rather on the bacteria lysis by the phage. Therefore, the main mechanism of action of the phage cocktail is bactericidal activity.

These findings indicate that, while not fully predictive of murine in vivo outcomes, the in vitro Virulence Index associated with preliminary assays using this alternative model can provide valuable indications of treatment efficacy and represent a useful tool for guiding preclinical phage therapy development. However, validation of the proposed pipeline should be performed in future studies with larger sample sizes—a limitation encountered in this work during its execution—and with other bacterial species.

## Conclusions

The present study focused primarily on the issue that has been the target of discussions in recent works involving phage therapy: the discordance between the in vitro and in vivo realms of this field. The work demonstrated that there are strong indications that the Virulence Index parameter, constructed through a broth assay, can be an excellent therapeutic tool, since, as presented, it showed a strong correlation with the survival of *Galleria mellonella* larvae and was consistent with the results of mice treatment assays. This work, therefore, suggests a simple framework that can be extremely effective for conducting preclinical assays. Finally, to reinforce this generated hypothesis, its validation in further studies with a wider range and diversity of bacteria and bacteriophages is recommended.

## Supplementary Information

Supplementary Material 1.

Supplementary Material 2.

## Data Availability

All data generated or analyzed during this study are included in this published article. All phage genomes were submitted to GenBank. Accession numbers for all phage genomes are PX946874, PX946875, and PX968304 for phages ph9027, ph1461, and ph3678, respectively.

## Abbreviations

ST: Spot Test
EOP: Efficiency of Platting
v_1_: Local Virulence
V_P_: Virulence Index
HR: Host Range
MOI: Multiplicity of Infection
S_L_: *Galleria mellonella* larvae survival
CFU: Colonie Forming Unit
PFU: Plaque Forming Unit
OD: Optical Density
NBT: Nitro-Blue Tetrazolium
NAG: N-acetyl-β-glucosaminidase
MPO: Myeloperoxidase
