# Supplementary figures and images for: Comparison and correlation of in vitro and in vivo approaches for determining Pseudomonas aeruginosa bacteriophages activity
Source: BMC Microbiol. 2026 Jun 13;26:631. doi: 10.1186/s12866-026-05289-w (PMC13374203; doi:10.1186/s12866-026-05289-w)

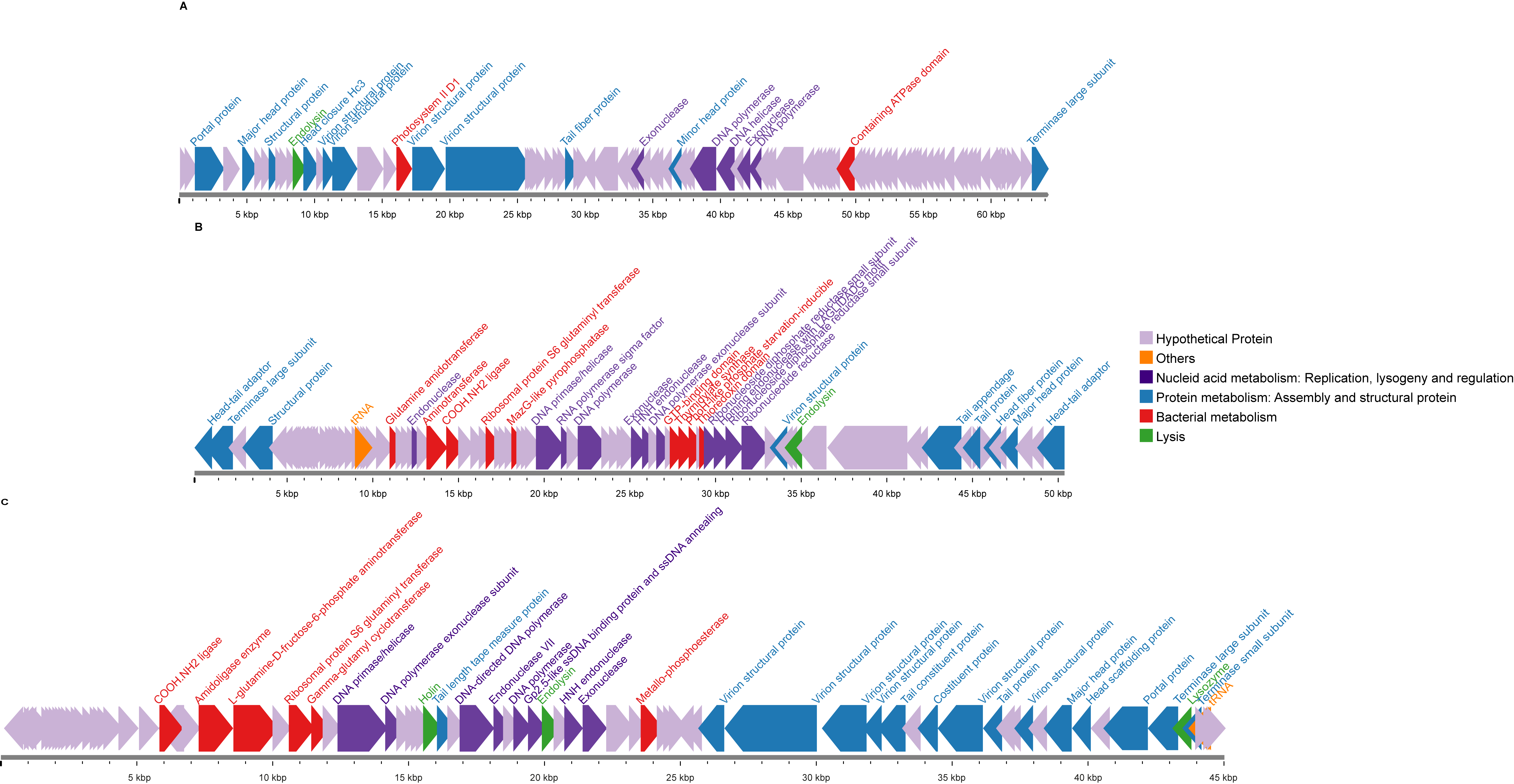

Supplement: Supplementary file 1 — Supplementary Material 1. [file 12866_2026_5289_MOESM1_ESM.png]
