# Supplementary material for: Comparison and correlation of in vitro and in vivo approaches for determining Pseudomonas aeruginosa bacteriophages activity
Source: BMC Microbiol. 2026 Jun 13;26:631. doi: 10.1186/s12866-026-05289-w (PMC13374203; doi:10.1186/s12866-026-05289-w)
Supplement: Supplementary file 2 — Supplementary Material 2. [file 12866_2026_5289_MOESM2_ESM.docx]

Supplementary Material

| **Supplementary Table S1. Characteristics of the clinical isolates used in this work** | | |
| --- | --- | --- |
| **Bacteria** | **Source** | **Antimicrobial resistance** |
| Pa9027 | Reference strain | - |
| Pa3400 | Tracheal secretion | - |
| PaJ704914 | Tracheal secretion | TZP, CZA, C/T, IMP/REL, CAZ, FEP, ATM, DOR, IMP, MEM, GEN, TOB, AMK, CIP, LEV, NOR |
| Pa2491 | Urine | TZP, CZA, IMP/REL, CAZ, FEP, ATM, DOR, IMP, MEM, GEN, TOB, AMK, CIP, LEV, NOR |
| Pa524 | Tracheal secretion | AMK |
| Pa01 | Reference strain | - |
| Pa1461 | Urine | TZP, C/T, IMP/REL, CAZ, FEP, ATM, DOR, IMP, MEM, GEN, TOB, AMK, CIP, LEV, NOR |
| Pa88718 | Tracheal secretion | TZP, IMI/REL, IMP, MEM |
| Pa66797 | Tracheal secretion | IMI/REL, FEP, IMP, MEM, GEN, TOB, AMK, CIP, LEV, NOR |
| Pa14078 | Tracheal secretion | FEP, ATM, DOR, IMP, GEN, TOB, AMK, CIP, LEV, NOR |
| Pa22459 | Tracheal secretion | TZP, CZA, C/T, IMP/REL, CAZ, FEP, ATM, DOR, IMP, MEM, GEN, TOB, AMK, CIP, LEV, NOR |
| Pa01556 | Urine | TZP, CZA, IMP/REL, CAZ, FEP, ATM, DOR, IMP, MEM, GEN, TOB, AMK, CIP, LEV, NOR |
| Pa3678 | Tracheal secretion | TZP, CZA, C/T, IMP/REL, CAZ, FEP, ATM, DOR, IMP, MEM, GEN, TOB, AMK, CIP, LEV, NOR |
| Pa49626 | Tracheal secretion | TZP, CZA, FEP, ATM, DOR, IMP, MEM, GEN, TOB, AMK, CIP, LEV, NOR |
| Pa96039 | Tracheal secretion | FEP, IMP, MEM, GEN, TOB, AMK, CIP, LEV, NOR |
| Pa21091 | Blood | ATM |
| Pa12071 | Tissue | ATM, LEV |
| Pa27853 | Reference strain | - |

ATM: Aztreonam; FEP: Cefepime; CAZ: Ceftazidime; CIP: Ciprofloxacin; GEN: Gentamicin; IMP: Imipenem; LEV: Levofloxacin; MEM: Meropenem; TZP: Piperacycline

Tazobactam; TOB: Tobramycin; CZA: Ceftazidime-Avibactam; IMP/REL: Imipenem-Relebactam; C/T: Ceftolozane-Tazobactam, DOR: Doripenem; AMK: Amikacin; NOR: Norfloxacin

| Supplementary Table S2. Functional genome annotation of ph9027 phage | | | | | | | | | |
| --- | --- | --- | --- | --- | --- | --- | --- | --- | --- |
| Prokka | | | **Pharokka** | | | **PhaBOX** | | | **Interpretation** |
| Genomic coordinates | **Strand** | **Predicted Protein** | **Genomic coordinates** | **Strand** | **Predicted Protein** | **Genomic coordinates** | **Strand** | **Predicted Protein** |  |
| 1..669 | + | hypothetical protein |  |  |  | 1..669 | + | hypothetical protein | hypothetical protein |
|  |  |  | 3..125 | + | hypothetical protein |  |  |  | hypothetical protein |
|  |  |  | 154..669 | + | hypothetical protein |  |  |  | hypothetical protein |
| 683..2869 | + | hypothetical protein | 683..2869 | + | portal protein | 683..2869 | + | portal protein | portal protein |
| 2862..4031 | + | hypothetical protein | 2802..4031 | + | hypothetical protein | 2862..4031 | + | hypothetical protein | hypothetical protein |
| 4213..5343 | + | major capsid protein | 4213..5343 | + | major head protein | 4213..5343 | + | major head protein | major head protein |
| 5380..5811 | + | hypothetical protein | 5380..5811 | + | hypothetical protein | 5380..5811 | + | hypothetical protein | hypothetical protein |
| 5889..6338 | + | hypothetical protein | 5889..6338 | + | hypothetical protein | 5889..6338 | + | hypothetical protein | hypothetical protein |
| 6343..7002 | + | hypothetical protein | 6343..7002 | + | structural protein | 6343..7002 | + | structural protein | structural protein |
| 7011..7310 | + | hypothetical protein | 7011..7310 | + | hypothetical protein | 7011..7310 | + | hypothetical protein | hypothetical protein |
| 7320..7631 | + | hypothetical protein | 7320..7631 | + | hypothetical protein | 7320..7631 | + | hypothetical protein | hypothetical protein |
| 7884..8159 | + | hypothetical protein | 7824..8159 | + | hypothetical protein | 7884..8159 | + | hypothetical protein | hypothetical protein |
| 8146..8769 | + | endolysin A | 8146..8769 | + | endolysin | 8146..8769 | + | endolysin | endolysin |
| 8766..9962 | + | hypothetical protein | 8766..9962 | + | head closure Hc3 | 8766..9962 | + | head closure Hc3 | Head closure Hc3 |
| 9952..10467 | + | hypothetical protein | 9952..10467 | + | hypothetical protein | 9952..10467 | + | hypothetical protein | hypothetical protein |
| 10478..10921 | + | hypothetical protein | 10478..10921 | + | virion structural protein | 10478..10921 | + | virion structural protein | virion structural protein |
| 10921..12783 | + | hypothetical protein | 10921..12783 | + | virion structural protein | 10921..12783 | + | virion structural protein | virion structural protein |
| 12783..14732 | + | hypothetical protein | 12783..14732 | + | hypothetical protein | 12783..14732 | + | hypothetical protein | hypothetical protein |
| 14734..15690 | + | hypothetical protein | 14734..15690 | + | hypothetical protein | 14734..15690 | + | hypothetical protein | hypothetical protein |
| 15690..16859 | + | hypothetical protein | 15690..16859 | + | photosystem II D1 | 15690..16859 | + | photosystem II D1 | photosystem II D1 |
| 16874..19303 | + | hypothetical protein | 16874..19303 | + | virion structural protein | 16874..19303 | + | virion structural protein | virion structural protein |
| 19361..25714 | + | hypothetical protein | 19361..25714 | + | virion structural protein | 19361..25714 | + | virion structural protein | virion structural protein |
|  |  |  | 25683..25820 | + | hypothetical protein |  |  |  | hypothetical protein |
|  |  |  | 25827..25988 | + | hypothetical protein |  |  |  | hypothetical protein |
|  |  |  | 26053..26148 | + | hypothetical protein |  |  |  | hypothetical protein |
|  |  |  | 26186..26284 | + | hypothetical protein |  |  |  | hypothetical protein |
| 26281..26874 | + | hypothetical protein | 26281..26874 | + | hypothetical protein | 26281..26874 | + | hypothetical protein | hypothetical protein |
| 26871..27548 | + | hypothetical protein | 26871..27548 | + | hypothetical protein | 26871..27548 | + | hypothetical protein | hypothetical protein |
| 27550..28065 | + | hypothetical protein | 27550..28065 | + | hypothetical protein | 27550..28065 | + | hypothetical protein | hypothetical protein |
| 28074..28280 | + | hypothetical protein | 28074..28280 | + | hypothetical protein | 28074..28280 | + | hypothetical protein | hypothetical protein |
| 28273..29205 | + | hypothetical protein | 28273..29205 | + | tail fiber protein | 28273..29205 | + | tail fiber protein | tail fiber protein |
| 29205..29507 | + | hypothetical protein | 29205..29507 | + | hypothetical protein | 29205..29507 | + | hypothetical protein | hypothetical protein |
| 29504..29731 | + | hypothetical protein | 29504..29731 | + | hypothetical protein | 29504..29731 | + | hypothetical protein | hypothetical protein |
| 29810..30199 | - | hypothetical protein | 29810..30199 | - | hypothetical protein | 29810..30199 | - | hypothetical protein | hypothetical protein |
| 30196..32184 | - | hypothetical protein | 30196..32184 | - | hypothetical protein | 30196..32184 | - | hypothetical protein | hypothetical protein |
| 32181..32708 | - | hypothetical protein | 32181..32708 | - | hypothetical protein | 32181..32708 | - | hypothetical protein | hypothetical protein |
| 32712..32882 | - | hypothetical protein | 32712..33020 | - | hypothetical protein | 33131..33373 | - | hypothetical protein | hypothetical protein |
| 33131..33373 | - | hypothetical protein | 33011..33109 | - | hypothetical protein | 33370..33909 | - | putative exonuclease | Exonuclease |
| 33370..33909 | - | hypothetical protein | 33131..33373 | - | hypothetical protein |  |  |  | hypothetical protein |
| 33909..34415 | - | hypothetical protein | 33370..33909 | - | exonuclease | 33909..34415 | - | hypothetical protein | hypothetical protein |
|  |  |  | 33909..34343 | - | hypothetical protein |  |  |  | hypothetical protein |
| 34475..34966 | - | hypothetical protein | 34475..35020 | - | hypothetical protein | 34475..34966 | - | hypothetical proteinn | hypothetical protein |
| 34905..35243 | - | hypothetical protein | 34905..35243 | - | hypothetical protein | 34905..35243 | - | hypothetical proteinn | hypothetical protein |
| 35260..36138 | - | hypothetical protein | 35260..36138 | - | hypothetical protein | 35260..36138 | - |  | hypothetical protein |
| 36207..36611 | - | hypothetical protein | 36207..36665 | - | minor head protein | 36207..36611 | - | minor head protein | minor head protein |
| 36634..37026 | - | hypothetical protein | 36634..37026 | - | hypothetical protein | 36634..37026 | - | hypothetical protein | hypothetical protein |
| 37001..37312 | - | hypothetical protein | 37001..37312 | - | hypothetical protein | 37001..37312 | - | hypothetical protein | hypothetical protein |
| 37309..37530 | - | hypothetical protein | 37309..37530 | - | hypothetical protein | 37309..37530 | - | hypothetical protein | hypothetical protein |
| 37540..39495 | - | hypothetical protein | 37540..39504 | - | DNA polymerase | 37540..39504 | - | DNA polymerase | DNA polymerase |
| 39515..40876 | - | hypothetical protein | 39515..40876 | - | DNA helicase | 39515..40876 | - | hypothetical protein | DNA helicase |
| 40886..41191 | - | hypothetical protein | 40886..41191 | - | hypothetical protein | 40886..41191 | - | hypothetical protein | hypothetical protein |
| 41188..41916 | - | hypothetical protein | 41188..41916 | - | exonuclease | 41188..41916 | - | hypothetical protein | hypothetical protein |
| 42005..42766 | - | hypothetical protein | 42005..42766 | - | DNA polymerase | 42005..42766 | - | DNA polymerase | DNA polymerase |
| 42776..43048 | - | hypothetical protein | 42776..43048 | - | hypothetical protein | 42776..43048 | - | hypothetical protein | hypothetical protein |
| 43029..43367 | - | hypothetical protein | 43029..43382 | - | hypothetical protein | 43029..43367 | - | hypothetical protein | hypothetical protein |
| 43364..43591 | - | hypothetical protein | 43364..43591 | - | hypothetical protein | 43364..43591 | - | hypothetical protein | hypothetical protein |
| 43588..45993 | - | hypothetical protein | 43588..45993 | - | hypothetical protein | 43588..45993 | - | hypothetical protein | hypothetical protein |
| 46136..46585 | - | hypothetical protein | 46136..46459 | - | hypothetical protein | 46136..46585 | - | hypothetical protein | hypothetical protein |
| 46588..46800 | - | hypothetical protein | 46588..46800 | - | hypothetical protein | 46588..46800 | - | hypothetical protein | hypothetical protein |
| 46797..47018 | - | hypothetical protein | 46797..47018 | - | hypothetical protein | 46797..47018 | - | hypothetical protein | hypothetical protein |
| 47042..47386 | - | hypothetical protein | 47042..47386 | - | hypothetical protein | 47042..47386 | - | hypothetical protein | hypothetical protein |
| 47394..47606 | - | hypothetical protein | 47394..47606 | - | hypothetical protein | 47394..47606 | - | hypothetical protein | hypothetical protein |
| 47616..47708 | - | hypothetical protein | 47616..47708 | - | hypothetical protein | 47616..47708 | - | hypothetical protein | hypothetical protein |
| 47705..48082 | - | hypothetical protein | 47705..48082 | - | hypothetical protein | 47705..48082 | - | hypothetical protein | hypothetical protein |
| 48069..48401 | - | hypothetical protein | 48069..48401 | - | hypothetical protein | 48069..48401 | - | hypothetical protein | hypothetical protein |
| 48459..49817 | - | hypothetical protein | 48459..49817 | - | hypothetical protein | 48459..49817 | - | containing ATPase domain | containing ATPase domain |
|  |  |  | 49837..49932 | - | hypothetical protein |  |  |  | hypothetical protein |
| 49992..50258 | - | hypothetical protein | 49992..50258 | - | hypothetical protein | 49992..50258 | - | hypothetical protein | hypothetical protein |
| 50255..50668 | - | hypothetical protein | 50255..50668 | - | hypothetical protein | 50255..50668 | - | hypothetical protein | hypothetical protein |
| 50649..51047 | - | hypothetical protein | 50649..51047 | - | hypothetical protein | 50649..51047 | - | hypothetical protein | hypothetical protein |
| 51059..51664 | - | hypothetical protein | 51059..51664 | - | hypothetical protein | 51059..51664 | - | hypothetical protein | hypothetical protein |
| 51640..51984 | - | hypothetical protein | 51640..51984 | - | hypothetical protein | 51640..51984 | - | hypothetical protein | hypothetical protein |
| 51988..52179 | - | hypothetical protein | 51988..52179 | - | hypothetical protein | 51988..52179 | - | hypothetical proteinn | hypothetical protein |
| 52179..52844 | - | hypothetical protein | 52179..52844 | - | hypothetical protein | 52179..52844 | - | hypothetical protein | hypothetical protein |
| 52847..53419 | - | hypothetical protein | 52847..53419 | - | hypothetical protein | 52847..53419 | - | hypothetical protein | hypothetical protein |
| 53412..53714 | - | hypothetical protein | 53412..53714 | - | hypothetical protein | 53412..53714 | - | hypothetical protein | hypothetical protein |
| 53711..53986 | - | hypothetical protein | 53711..54028 | - | hypothetical protein | 53711..53986 | - | hypothetical protein | hypothetical protein |
| 53983..54189 | - | hypothetical protein | 53983..54189 | - | hypothetical protein | 53983..54189 | - | hypothetical protein | hypothetical protein |
| 54186..54437 | - | hypothetical protein | 54186..54437 | - | hypothetical protein | 54186..54437 | - | hypothetical protein | hypothetical protein |
| 54440..54991 | - | hypothetical protein | 54440..54991 | - | hypothetical protein | 54440..54991 | - | hypothetical protein | hypothetical protein |
| 55020..55373 | - | hypothetical protein | 55020..55373 | - | hypothetical protein | 55020..55373 | - | hypothetical protein | hypothetical protein |
| 55385..55918 | - | hypothetical protein | 55385..55918 | - | hypothetical protein | 55385..55918 | - | hypothetical protein | hypothetical protein |
| 55923..56144 | - | hypothetical protein | 55923..56144 | - | hypothetical protein | 55923..56144 | - | hypothetical protein | hypothetical protein |
| 56148..57239 | - | hypothetical protein | 56148..57239 | - | hypothetical protein | 56148..57239 | - | hypothetical protein | hypothetical protein |
| 57301..57627 | - | hypothetical protein | 57301..57627 | - | hypothetical protein | 57301..57627 | - | hypothetical protein | hypothetical protein |
| 57639..57842 | - | hypothetical protein | 57639..57842 | - | hypothetical protein | 57639..57842 | - | hypothetical protein | hypothetical protein |
|  |  |  | 57981..58259 | - | hypothetical protein |  |  |  | hypothetical protein |
|  |  |  | 58307..58507 | - | hypothetical protein |  |  |  | hypothetical protein |
| 58520..58930 | - | hypothetical protein | 58520..58942 | - | hypothetical protein | 58520..58930 | - | hypothetical protein | hypothetical protein |
| 59017..59808 | - | hypothetical protein | 59017..59808 | - | hypothetical protein | 59017..59808 | - | hypothetical protein | hypothetical protein |
| 59885..60082 | - | hypothetical protein | 59885..60082 |  | hypothetical protein | 59885..60082 | - | hypothetical protein | hypothetical protein |
| 60079..60360 | - | hypothetical protein | 60079..60360 |  | hypothetical protein | 60079..60360 | - | hypothetical protein | hypothetical protein |
| 60429..60659 | - | hypothetical protein | 60429..60659 |  | hypothetical protein | 60429..60659 | - | hypothetical protein | hypothetical protein |
|  |  | hypothetical protein | 60701..61021 |  | hypothetical protein |  |  |  | hypothetical protein |
| 61018..61443 | + | hypothetical protein | 61018..61443 |  | hypothetical protein | 61018..61443 | + | hypothetical protein | hypothetical protein |
| 61450..61809 | + | hypothetical protein | 61450..61809 |  | hypothetical protein | 61450..61809 | + | hypothetical protein | hypothetical protein |
| 61815..62168 | + | hypothetical protein | 61815..62168 |  | hypothetical protein | 61815..62168 | + | hypothetical protein | hypothetical protein |
| 62316..63026 | + | hypothetical protein | 62316..63026 |  | hypothetical protein | 62316..63026 | + | hypothetical protein | hypothetical protein |
| 62887..63435 | - | hypothetical protein |  |  |  |  |  |  | hypothetical protein |
|  |  |  | 63007..64263 | - | terminase large subunit | 63007..64263 | + | terminase large subunit | terminase large subunit |
| 63475..64257 | - | hypothetical protein |  |  |  |  |  |  | hypothetical protein |

| Supplementary Table S3. Functional genome annotation of ph1461 phage | | | | | | | | | |
| --- | --- | --- | --- | --- | --- | --- | --- | --- | --- |
| Prokka | | | **Pharokka** | | | **PhaBOX** | | | **Interpretation** |
| Genomic coordinates | **Strand** | **Predicted Protein** | **Genomic coordinates** | **Strand** | **Predicted Protein** | **Genomic coordinates** | **Strand** | **Predicted Protein** |  |
|  |  |  | 1..213 | - | head-tail adaptor | 1..213 | - | hypothetical protein | head-tail adaptator |
| 226..1833 | - | hypothetical protein | 226..1833 | - | hypothetical protein | 226..1833 | - | putative terminase large subunit | terminase large subunit |
| 1837..2406 | - | hypothetical protein | 1837..2340 | - | hypothetical protein | 1837..2406 | - | hypothetical protein | hypothetical protein |
| 2390..4153 | - | hypothetical protein | 2390..4153 | - | hypothetical protein | 2390..4153 | - | structural protein | structural protein |
| 4150..4743 | - | hypothetical protein | 4150..4743 | - | hypothetical protein | 4150..4743 | - | hypothetical protein | hypothetical protein |
|  |  |  | 4740..4874 | - | hypothetical protein |  |  |  | hypothetical protein |
|  |  |  | 5031..5129 | - | hypothetical protein |  |  |  | hypothetical protein |
|  |  |  | 5143..5241 | - | hypothetical protein |  |  |  | hypothetical protein |
|  |  |  | 5245..5340 | - | hypothetical protein |  |  |  | hypothetical protein |
|  |  |  | 5391..5573 | - | hypothetical protein |  |  |  | hypothetical protein |
|  |  |  | 5674..5790 | - | hypothetical protein |  |  |  | hypothetical protein |
| 5843..5992 | + | hypothetical protein |  |  |  | 5843..5992 | + | hypothetical protein | hypothetical protein |
|  |  |  | 5896..5997 | - | hypothetical protein |  |  |  | hypothetical protein |
| 6016..6219 | + | hypothetical protein |  |  |  | 6016..6219 | + | hypothetical protein | hypothetical protein |
|  |  |  | 6045..6194 | - | hypothetical protein |  |  |  | hypothetical protein |
|  |  |  | 6208..6369 | - | hypothetical protein |  |  |  | hypothetical protein |
|  |  |  | 6402..6491 | + | hypothetical protein |  |  |  | hypothetical protein |
| 6575..6847 | + | hypothetical protein | 6575..6847 | + | hypothetical protein | 6575..6847 | + | hypothetical protein | hypothetical protein |
|  |  |  | 6920..7093 | + | hypothetical protein |  |  |  | hypothetical protein |
| 7141..7686 | + | hypothetical protein | 7141..7686 | + | hypothetical protein | 7141..7686 | + | hypothetical protein | hypothetical protein |
|  |  |  | 7667..7774 | + | hypothetical protein |  |  |  | hypothetical protein |
| 7821..8012 | + | hypothetical protein | 7821..8012 | + | hypothetical protein | 7821..8012 | + | hypothetical protein | hypothetical protein |
| 8009..8233 | + | hypothetical protein | 8009..8233 | + | hypothetical protein | 8009..8233 | + | hypothetical protein | hypothetical protein |
|  |  |  | 8211..8309 | + | hypothetical protein |  |  |  | hypothetical protein |
| 8373..8699 | + | hypothetical protein | 8319..8699 | + | hypothetical protein | 8373..8699 | + | hypothetical protein | hypothetical protein |
| 8692..9063 | + | hypothetical protein | 8692..9063 | + | hypothetical protein | 8692..9063 | + | hypothetical protein | hypothetical protein |
| 9134..9418 | + | hypothetical protein | 9134..9418 | + | hypothetical protein | 9134..9418 | + | hypothetical protein | hypothetical protein |
|  |  |  | 9427..9509 | + |  |  |  |  | hypothetical protein |
|  |  |  | 9517..9699 | + | hypothetical protein |  |  |  | hypothetical protein |
|  |  |  | 9740..9829 | + | hypothetical protein |  |  |  | hypothetical protein |
| 9834..10001 | + | hypothetical protein | 9834..10001 | + | hypothetical protein | 9834..10001 | + | hypothetical protein | hypothetical protein |
| 10001..10231 | + | hypothetical protein | 10001..10231 | + | hypothetical protein | 10001..10231 | + | hypothetical protein | hypothetical protein |
| 10233..11168 | + | hypothetical protein | 10233..11168 | + | hypothetical protein | 10233..11168 | + | hypothetical protein | hypothetical protein |
| 11161..11781 | + | hypothetical protein | 11206..11781 | + | glutamine amidotransferase | 11161..11781 | + | glutamine amidotransferase | glutamine amidotransferase |
| 11778..11900 | + | hypothetical protein | 11778..11900 | + | hypothetical protein | 11778..11900 | + | hypothetical protein | hypothetical protein |
| 11910..12413 | + | hypothetical protein | 11910..12413 | + | hypothetical protein | 11910..12413 | + | hypothetical protein | hypothetical protein |
|  |  |  | 12453..12560 | + | hypothetical protein |  |  |  | hypothetical protein |
| 12557..13003 | + | hypothetical protein | 12557..13003 | + | endonuclease | 12557..13003 | + | endonuclease | endonuclease |
|  |  |  | 13026..13130 | + | hypothetical protein |  |  |  | hypothetical protein |
| 13064..14353 | + | hypothetical protein | 13133..14353 | + | aminotransferase | 13064..14353 | + | aminotransferase | aminotransferase |
| 14363..15226 | + | hypothetical protein | 14363..15226 | + | COOH.NH2 ligase | 14363..15226 | + | COOH.NH2 ligase | COOH.NH2 ligase |
| 15228..15776 | + | hypothetical protein | 15207..15776 | + | hypothetical protein | 15228..15776 | + | hypothetical protein | hypothetical protein |
| 15773..16636 | + | hypothetical protein | 15773..16636 | + | amidoligase enzyme | 15773..16636 | + | amidoligase enzyme | amidoligase enzyme |
|  |  |  | 16641..16742 | + | hypothetical protein |  |  |  | hypothetical protein |
| 16720..17472 | + | hypothetical protein | 16720..17472 | + | ribosomal protein S6 glutaminyl transferase | 16720..17472 | + | ribosomal protein S6 glutaminyl transferase | ribosomal protein S6 glutaminyl transferase |
| 17469..17717 | + | hypothetical protein | 17469..17717 | + | hypothetical protein | 17469..17717 | + | hypothetical protein | hypothetical protein |
| 17717..18001 | + | hypothetical protein | 17717..18001 | + | hypothetical protein | 17717..18001 | + | hypothetical protein | hypothetical protein |
| 17994..18416 | + | hypothetical protein | 17994..18416 | + | hypothetical protein | 17994..18416 | + | hypothetical protein | hypothetical protein |
| 18449..18748 | + | hypothetical protein | 18449..18748 | + | MazG-like pyrophosphatase | 18449..18748 | + | MazG-like pyrophosphatase | MazG-like pyrophosphatase |
| 18741..19067 | + | hypothetical protein | 18714..19067 | + | hypothetical protein | 18741..19067 | + | hypothetical protein | hypothetical protein |
| 19064..19255 | + | hypothetical protein | 19064..19255 | + | hypothetical protein | 19064..19255 | + | hypothetical protein | hypothetical protein |
| 19255..19524 | + | hypothetical protein | 19255..19524 | + | hypothetical protein | 19255..19524 | + | hypothetical protein | hypothetical protein |
| 19525..21240 | + | DNA primase/helicase | 19525..21240 | + | DNA primase/helicase | 19525..21240 | + | DNA primase/helicase | DNA primase/helicase |
| 21240..21740 | + | hypothetical protein | 21240..21740 | + | RNA polymerase sigma factor | 21240..21740 | + | RNA polymerase sigma factor | RNA polymerase sigma factor |
| 21733..21903 | + | hypothetical protein | 21733..21903 | + | hypothetical protein | 21733..21903 | + | hypothetical protein | hypothetical protein |
| 21896..23752 | + | hypothetical protein | 21962..23752 | + | DNA polymerase | 21896..23752 | + | putative DNA polymerase | DNA polymerase |
| 23764..23928 | + | hypothetical protein | 23764..23928 | + | hypothetical protein | 23764..23928 | + | hypothetical protein | hypothetical protein |
| 23928..24656 | + | hypothetical protein | 23928..24656 | + | hypothetical protein | 23928..24656 | + | hypothetical protein | hypothetical protein |
| 24650..24823 | + | hypothetical protein | 24650..24823 | + | hypothetical protein | 24650..24823 | + | hypothetical protein | hypothetical protein |
| 24816..25121 | + | hypothetical protein | 24816..25121 | + | hypothetical protein | 24816..25121 | + | hypothetical protein | hypothetical protein |
| 25148..26011 | + | hypothetical protein | 25148..26011 | + | exonuclease | 25148..26011 | + | hypothetical protein | hypothetical protein |
| 26011..26436 | + | hypothetical protein | 26011..26436 | + | HNH endonuclease | 26011..26436 | + | HNH endonuclease | HNH endonuclease |
| 26426..26677 | + | hypothetical protein | 26507..26677 | + | hypothetical protein | 26426..26677 | + | hypothetical protein | hypothetical protein |
| 26670..27437 | + | hypothetical protein | 26670..27437 | + | DNA polymerase exonuclease subunit | 26670..27437 | + | DNA polymerase exonuclease subunit | DNA polymerase exonuclease subunit |
|  |  |  | 27439..27666 | + | hypothetical protein |  |  |  | hypothetical protein |
| 27666..28013 | + | hypothetical protein | 27666..28013 | + | GTP-binding domain | 27666..28013 | + | DNA recombination-mediator protein A | GTP-binding domain |
| 28010..28684 | + | putative flavin-dependent thymidylate synthase | 28010..28684 | + | thymidylate synthase | 28010..28684 | + | thymidylate synthase | thymidylate synthase |
| 28677..29324 | + | hypothetical protein | 28563..29324 | + | PhoH-like phosphate starvation-inducible | 28677..29324 | + | PhoH-like phosphate starvation-inducible | PhoH-like phosphate starvation-inducible |
| 29308..29463 | + | hypothetical protein | 29308..29463 | + | hypothetical protein | 29308..29463 | + | hypothetical protein | hypothetical protein |
| 29435..29683 | + | hypothetical protein | 29435..29683 | + | thioredoxin domain | 29435..29683 | + | hypothetical protein | thioredoxin domain |
| 29667..29990 | + | hypothetical protein | 29667..29990 | + | ribonucleoside diphosphate reductase small subunit | 29667..29990 | + | hypothetical protein | ribonucleoside diphosphate reductase small subunit |
| 30092..30742 | + | hypothetical protein | 30074..30742 | + | homing endonuclease with LAGLIDADG motif | 30092..30742 | + | hypothetical protein | homing endonuclease with LAGLIDADG motif |
| 30746..31450 | + | hypothetical protein | 30746..31450 | + | ribonucleoside diphosphate reductase small subunit | 30746..31450 | + | ribonucleotide reductase | ribonucleoside diphosphate reductase small subunit |
| 31447..33138 | + | Ribonucleoside-diphosphate reductase large subunit | 31519..33138 | + | ribonucleotide reductase | 31447..33138 | + | ribonucleotide reductase | ribonucleotide reductase |
| 33274..33495 | + | hypothetical protein | 33274..33495 | + | hypothetical protein | 33274..33495 | + | hypothetical protein | hypothetical protein |
| 33537..33815 | - | hypothetical protein | 33537..33815 | - | virion structural protein | 33537..33815 | - | virion structural protein | virion structural protein |
| 33815..34174 | - | hypothetical protein | 33815..34174 | - | hypothetical protein | 33815..34174 | - | hypothetical protein | hypothetical protein |
| 34176..34322 | - | hypothetical protein | 34176..34322 | - | hypothetical protein | 34176..34322 | - | hypothetical protein | hypothetical protein |
| 34324..34761 | - | Endolysin | 34324..34761 | - | endolysin | 34324..34761 | - | endolysin | endolysin |
| 34733..36526 | - | hypothetical protein | 34733..36472 | - | hypothetical protein | 34733..36526 | - | hypothetical protein | hypothetical protein |
| 36526..41190 | - | hypothetical protein | 36526..41190 | - | hypothetical protein | 36526..41190 | - | hypothetical protein | hypothetical protein |
| 41201..41758 | - | hypothetical protein | 41201..41758 | - | hypothetical protein | 41201..41758 | - | hypothetical protein | hypothetical protein |
| 41719..42048 | - | hypothetical protein | 41719..42048 | - | hypothetical protein | 41719..42048 | - | hypothetical protein | hypothetical protein |
| 42035..44350 | - | hypothetical protein | 42035..44350 | - | tail appendage | 42035..44350 | - | tail appendage | tail appendage |
| 44334..44522 | - | hypothetical protein | 44334..44522 | - | hypothetical protein | 44334..44522 | - | hypothetical protein | hypothetical protein |
| 44532..45335 | - | hypothetical protein | 44532..45335 | - | tail protein | 44532..45335 | - | tail protein | tail protein |
|  |  |  | 45362..45652 | + | hypothetical protein |  |  |  | hypothetical protein |
| 45337..45957 | - | hypothetical protein |  |  |  | 45337..45957 | - | hypothetical protein | hypothetical protein |
|  |  |  | 45743..45838 | + | hypothetical protein |  |  |  | hypothetical protein |
|  |  |  | 45877..46005 | + | hypothetical protein |  |  |  | hypothetical protein |
| 46011..46277 | - | hypothetical protein | 46011..46277 | - | head fiber protein | 46011..46277 | - | head fiber protein | head fiber protein |
| 46288..46644 | - | hypothetical protein | 46288..46644 | - | hypothetical protein | 46288..46644 | - | hypothetical protein | hypothetical protein |
| 46658..47599 | - | hypothetical protein | 46658..47599 | - | major head protein | 46658..47599 | - | hypothetical protein | major head protein |
| 47610..48368 | - | hypothetical protein | 47610..48368 | - | hypothetical protein | 47610..48368 | - | hypothetical protein | hypothetical protein |
| 48511..48768 | - | hypothetical protein | 48511..48768 | - | hypothetical protein | 48511..48768 | - | hypothetical protein | hypothetical protein |
| 48768..50270 | - | hypothetical protein | 48768..50363 | - | head-tail adaptor | 48768..50363 | - | head-tail adaptor | head-tail adaptor |

| Supplementary Table S4. Functional genome annotation of ph3678 phage | | | | | | | | | |
| --- | --- | --- | --- | --- | --- | --- | --- | --- | --- |
| Prokka | | | **Pharokka** | | | **PhaBOX** | | | **Interpretation** |
| Genomic coordinates | **Strand** | **Predicted Protein** | **Genomic coordinates** | **Strand** | **Predicted Protein** | **Genomic coordinates** | **Strand** | **Predicted Protein** |  |
|  |  |  | 371..72 | - | hypothetical protein |  |  |  | hypothetical protein |
| 379..597 | - | hypothetical protein | 597..379 | - | hypothetical protein | 597..379 | - | hypothetical protein | hypothetical protein |
|  |  |  | 770..597 | - | hypothetical protein |  |  |  | hypothetical protein |
|  |  |  | 903..814 | - | hypothetical protein |  |  |  | hypothetical protein |
| 1019..1324 | + | hypothetical protein | 1019..1324 | + | hypothetical protein | 1019..1324 | + | hypothetical protein | hypothetical protein |
| 1328..1810 | + | hypothetical protein | 1328..1810 | + | hypothetical protein | 1328..1810 | + | hypothetical protein | hypothetical protein |
| 1885..2127 | + | hypothetical protein | 1885..2127 | + | hypothetical protein | 1885..2127 | + | hypothetical protein | hypothetical protein |
|  |  |  | 2138..2239 | + | hypothetical protein | 2417..2599 | + | hypothetical protein | hypothetical protein |
|  |  |  | 2292..2420 | + | hypothetical protein |  |  |  | hypothetical protein |
| 2417..2599 | + | hypothetical protein |  |  |  |  |  |  | hypothetical protein |
|  |  |  | 2453..2599 | + | hypothetical protein |  |  |  | hypothetical protein |
|  |  |  | 2599..2721 | + | hypothetical protein |  |  |  | hypothetical protein |
| 2734..2889 | + | hypothetical protein | 2734..2889 | + | hypothetical protein | 2734..2889 | + | hypothetical protein | hypothetical protein |
| 2876..3022 | + | hypothetical protein | 2876..3022 | + | hypothetical protein | 2876..3022 | + | hypothetical protein | hypothetical protein |
| 3032..3289 | + | hypothetical protein | 3032..3289 | + | hypothetical protein | 3032..3289 | + | hypothetical protein | hypothetical protein |
| 3252..3560 | + | hypothetical protein | 3252..3560 | + | hypothetical protein | 3252..3560 | + | hypothetical protein | hypothetical protein |
|  |  |  | 3533..3628 | + | hypothetical protein |  |  |  | hypothetical protein |
|  |  |  | 3687..4244 | + | hypothetical protein |  |  |  | hypothetical protein |
| 3819..4244 | + | hypothetical protein | 4272..4361 | + | hypothetical protein | 3819..4244 | + | hypothetical protein | hypothetical protein |
| 4370..4582 | + | hypothetical protein | 4370..4582 | + | hypothetical protein | 4370..4582 | + | hypothetical protein | hypothetical protein |
| 4575..4796 | + | hypothetical protein | 4575..4796 | + | hypothetical protein | 4575..4796 | + | hypothetical protein | hypothetical protein |
|  |  |  | 4803..5132 | + | hypothetical protein |  |  |  | hypothetical protein |
| 5029..5157 | + | hypothetical protein |  |  |  | 5029..5157 | + | hypothetical protein | hypothetical protein |
| 5231..6193 | + | hypothetical protein | 5231..6193 | + | hypothetical protein | 5231..6193 | + | hypothetical protein | hypothetical protein |
| 6212..7171 | + | hypothetical protein | 6212..7171 | + | hypothetical protein | 6212..7171 | + | hypothetical protein | hypothetical protein |
|  |  |  | 8007..7126 | - | hypothetical protein |  |  |  | hypothetical protein |
| 7168..7968 | + | hypothetical protein |  |  |  | 7168..7968 | + | COOH.NH2 ligase | COOH.NH2 ligase |
|  |  |  | 7961..8530 | + | hypothetical protein |  |  |  | hypothetical protein |
| 8006..8530 | + | hypothetical protein |  |  |  | 8006..8530 | + | hypothetical protein | hypothetical protein |
| 8506..9681 | + | hypothetical protein | 8506..9681 | + | amidoligase enzyme | 8506..9681 | + | amidoligase enzyme | amidoligase enzyme |
| 9693..11225 | + | hypothetical protein | 9693..11225 | + | L-glutamine-D-fructose-6-phosphate aminotransferase | 9693..11225 | + | L-glutamine-D-fructose-6-phosphate aminotransferase | L-glutamine-D-fructose-6-phosphate aminotransferase |
| 11235..11456 | + | hypothetical protein | 11235..11456 | + | hypothetical protein | 11235..11456 | + | hypothetical protein | hypothetical protein |
| 11587..12471 | + | hypothetical protein | 11587..12471 | + | ribosomal protein S6 glutaminyl transferase | 11587..12471 | + | ribosomal protein S6 glutaminyl transferase | ribosomal protein S6 glutaminyl transferase |
| 12471..12869 | + | hypothetical protein | 12471..12869 | + | gamma-glutamyl cyclotransferase | 12471..12869 | + | gamma-glutamyl cyclotransferase | gamma-glutamyl cyclotransferase |
| 12869..13246 | + | hypothetical protein | 12869..13246 | + | hypothetical protein | 12869..13246 | + | hypothetical protein | hypothetical protein |
| 13247..14956 | + | DNA primase/helicase | 13247..14956 | + | DNA primase/helicase | 13247..14956 | + | DNA primase/helicase | DNA primase/helicase |
| 14940..15449 | + | hypothetical protein | 14940..15449 | + | hypothetical protein | 14940..15449 | + | DNA polymerase exonuclease subunit | DNA polymerase exonuclease subunit |
| 15416..15727 | + | hypothetical protein | 15416..15727 | + | hypothetical protein | 15416..15727 | + | hypothetical protein | hypothetical protein |
| 15763..15996 | + | hypothetical protein | 15763..15996 | + | hypothetical protein | 15763..15996 | + | hypothetical protein | hypothetical protein |
| 15977..16168 | + | hypothetical protein | 15977..16168 | + | hypothetical protein | 15977..16168 | + | hypothetical protein | hypothetical protein |
| 16158..16319 | + | hypothetical protein | 16158..16319 | + | hypothetical protein | 16158..16319 | + | hypothetical protein | hypothetical protein |
| 16350..16628 | + | hypothetical protein | 16350..16628 | + | holin | 16350..16628 | + | holin | holin |
| 16705..17214 | + | hypothetical protein | 16705..17214 | + | tail length tape measure protein | 16705..17214 | + | tail length tape measure protein | tail length tape measure protein |
|  |  |  | 17219..17416 | + | hypothetical protein |  |  |  | hypothetical protein |
| 17243..17416 | + | hypothetical protein |  |  |  | 17243..17416 | + | hypothetical protein | hypothetical protein |
| 17417..18598 | + | DNA-directed DNA polymerase |  |  |  | 17417..18598 | + | DNA polymerase part II | DNA-directed DNA polymerase |
|  |  |  | 17582..18598 | + | hypothetical protein |  |  |  | hypothetical protein |
| 18701..19084 | + | hypothetical protein | 18701..19084 | + | endonuclease VII | 18701..19084 | + | endonuclease VII | endonuclease VII |
|  |  |  | 19086..19349 | + | hypothetical protein |  |  |  | hypothetical protein |
| 19415..19723 | + | hypothetical protein | 19415..19723 | + | DNA polymerase | 19415..19723 | + | DNA polymerase | DNA polymerase |
| 19793..20347 | + | hypothetical protein | 19793..20347 | + | Gp2.5-like ssDNA binding protein and ssDNA annealing protein | 19793..20347 | + | Gp2.5-like ssDNA binding protein and ssDNA annealing protein | Gp2.5-like ssDNA binding protein and ssDNA annealing protein |
|  |  |  | 20259..20828 | + | endolysin; inhibits RNA polymerase | 20325..20828 | + | endolysin; inhibits RNA polymerase | endolysin |
| 20325..20828 | + | hypothetical protein |  |  |  |  |  |  | hypothetical protein |
| 20800..21084 | + | hypothetical protein | 20800..21084 | + | hypothetical protein | 20800..21084 | + | hypothetical protein | hypothetical protein |
| 21084..21533 | + | hypothetical protein | 21084..21533 | + | endonuclease | 21084..21533 | + | HNH endonuclease | HNH endonuclease |
| 21618..22448 | + | hypothetical protein | 21618..22448 | + | exonuclease | 21618..22448 | + | exonuclease | exonuclease |
| 22423..23376 | + | hypothetical protein | 22423..23376 | + | hypothetical protein | 22423..23376 | + | hypothetical protein | hypothetical protein |
|  |  |  | 23426..23608 | + | endonuclease |  |  |  | endonuclease |
| 23601..24362 | + | hypothetical protein | 23601..24362 | + | metallo-phosphoesterase | 23601..24362 | + | hypothetical protein | metallo-phosphoesterase |
| 24359..24577 | + | hypothetical protein | 24359..24577 | + | hypothetical protein | 24359..24577 | + | hypothetical protein | hypothetical protein |
| 24581..24790 | + | hypothetical protein | 24581..24790 | + | hypothetical protein | 24581..24790 | + | hypothetical protein | hypothetical protein |
| 24777..24983 | + | hypothetical protein | 24777..24983 | + | hypothetical protein | 24777..24983 | + | hypothetical protein | hypothetical protein |
| 24961..25056 | + | hypothetical protein |  |  |  | 24961..25056 | + | - | hypothetical protein |
|  |  |  | 25038..25139 | - | hypothetical protein |  |  |  | hypothetical protein |
| 25191..25547 | - | hypothetical protein | 25191..25547 | - | hypothetical protein | 25191..25547 | - | hypothetical protein | hypothetical protein |
| 25562..26449 | - | hypothetical protein | 25562..26449 | - | virion structural protein | 25562..26449 | - | virion structural protein | virion structural protein |
| 26461..29631 | - | hypothetical protein | 26461..29613 | - | virion structural protein | 26461..29631 | - | virion structural protein | virion structural protein |
| 29787..31310 | - | hypothetical protein | 29787..31310 | - | virion structural protein | 29787..31310 | - | virion structural protein | virion structural protein |
| 31315..31698 | - | hypothetical protein | 31315..31698 | - | virion structural protein | 31315..31698 | - | virion structural protein | virion structural protein |
| 31698..32642 | - | hypothetical protein | 31698..32642 | - | hypothetical protein | 31698..32642 | - | putative tail constituent protein | putative tail constituent protein |
| 32623..33057 | - | hypothetical protein | 32623..33057 | - | hypothetical protein | 32623..33057 | - | hypothetical protein | hypothetical protein |
| 33054..33743 | - | hypothetical protein | 33054..33743 | - | hypothetical protein | 33054..33743 | - | constituent protein | constituent protein |
| 33740..35281 | - | hypothetical protein | 33740..35281 | - | virion structural protein | 33740..35281 | - | virion structural protein | virion structural protein |
| 35290..35937 | - | hypothetical protein | 35290..35937 | - | tail protein | 35290..35937 | - | tail protein | tail protein |
| 35927..36175 | - | hypothetical protein | 35927..36175 | - | hypothetical protein | 35927..36175 | - | hypothetical protein | hypothetical protein |
| 36159..36350 | - | hypothetical protein | 36159..36350 | - | hypothetical protein | 36159..36350 | - | hypothetical protein | hypothetical protein |
| 36361..36987 | - | hypothetical protein | 36361..36987 | - | virion structural protein | 36361..36987 | - | virion structural protein | virion structural protein |
| 36991..37311 | - | hypothetical protein | 36991..37311 | - | hypothetical protein | 36991..37311 | - | hypothetical protein | hypothetical protein |
| 37359..38312 | - | Major capsid protein | 37359..38312 | - | major head protein | 37359..38312 | - | major head protein | major head protein |
| 38331..39323 | - | hypothetical protein | 38331..39323 | - | head scaffolding protein | 38331..39323 | - | head scaffolding protein | head scaffolding protein |
|  |  |  | 39323..39514 | - | hypothetical protein |  |  |  | hypothetical protein |
| 39323..39565 | - | hypothetical protein |  |  |  | 39323..39565 | - | hypothetical protein | hypothetical protein |
| 39568..41688 | - | hypothetical protein | 39568..41688 | - | portal protein | 39568..41688 | - | portal protein | portal protein |
| 41688..43136 | - | Terminase, large subunit | 41688..43136 | - | hypothetical protein | 41688..43136 | - | terminase large subunit | terminase large subunit |
| 43136..43477 | - | hypothetical protein | 43136..43498 | - | hypothetical protein | 43136..43477 | - | lysozyme | lysozyme |
| 43566..44024 | - | hypothetical protein | 43566..44024 | - | terminase small subunit | 43566..44024 | - | terminase small subunit | terminase small subunit |
| 44047..44123 | - | tRNA-Pro(tgg) |  |  |  |  |  |  | tRNA-Pro(tgg) |
| 44128..44203 | - | tRNA-Asp(gtc) |  |  |  |  |  |  | tRNA-Asp(gtc) |
| 44208..44283 | - | tRNA-Asn(gtt) |  |  |  |  |  |  | tRNA-Asn(gtt) |
|  |  |  | 44354..44455 | + | hypothetical protein |  |  |  | hypothetical protein |
|  |  |  | 44742..44843 | + | hypothetical protein |  |  |  | hypothetical protein |
|  |  |  | 44892..45014 | + | hypothetical protein |  |  |  | hypothetical protein |
